# Carbon cycle inverse modeling suggests large changes in fractional organic burial are consistent with the carbon isotope record and may have contributed to the rise of oxygen

**DOI:** 10.1111/gbi.12440

**Published:** 2021-03-25

**Authors:** Joshua Krissansen‐Totton, Michael A. Kipp, David C. Catling

**Affiliations:** ^1^ Department of Earth and Space Sciences/Astrobiology Program University of Washington Seattle WA USA; ^2^ Virtual Planetary Laboratory NASA Nexus for Exoplanet System Science Seattle WA USA; ^3^ Department of Astronomy and Astrophysics University of California Santa Cruz CA USA; ^4^ Division of Geological and Planetary Sciences California Institute of Technology Pasadena CA USA

**Keywords:** carbon cycle, carbon isotopes, organic burial, oxygen, Precambrian, weathering

## Abstract

Abundant geologic evidence shows that atmospheric oxygen levels were negligible until the Great Oxidation Event (GOE) at 2.4–2.1 Ga. The burial of organic matter is balanced by the release of oxygen, and if the release rate exceeds efficient oxygen sinks, atmospheric oxygen can accumulate until limited by oxidative weathering. The organic burial rate relative to the total carbon burial rate can be inferred from the carbon isotope record in sedimentary carbonates and organic matter, which provides a proxy for the oxygen source flux through time. Because there are no large secular trends in the carbon isotope record over time, it is commonly assumed that the oxygen source flux changed only modestly. Therefore, declines in oxygen sinks have been used to explain the GOE. However, the average isotopic value of carbon fluxes into the atmosphere–ocean system can evolve due to changing proportions of weathering and outgassing inputs. If so, large secular changes in organic burial would be possible despite unchanging carbon isotope values in sedimentary rocks. Here, we present an inverse analysis using a self‐consistent carbon cycle model to determine the maximum change in organic burial since ~4 Ga allowed by the carbon isotope record and other geological proxies. We find that fractional organic burial may have increased by 2–5 times since the Archean. This happens because O_2_‐dependent continental weathering of ^13^C‐depleted organics changes carbon isotope inputs to the atmosphere–ocean system. This increase in relative organic burial is consistent with an anoxic‐to‐oxic atmospheric transition around 2.4 Ga without declining oxygen sinks, although these likely contributed. Moreover, our inverse analysis suggests that the Archean absolute organic burial flux was comparable to modern, implying high organic burial efficiency and ruling out very low Archean primary productivity.

**Key Points**

- Oxygen‐dependent organic weathering and preferential subduction of organics can decouple the isotope record from organic burial, potentially explaining the disagreement between modern fractional organic burial (~0.3) and the organic fraction of crustal carbon (~0.15).
- The absolute organic burial flux in the Archean was probably comparable to today. This rules out extremely low estimates of Archean productivity and suggests organic burial efficiency was at least an order of magnitude higher than modern burial efficiency.
- Organic burial relative to total carbon burial could have increased by 2–5 times (1σ) over Earth history.
- This increase in organic burial is consistent with the carbon isotope record and may have contributed to the transition from an anoxic‐to‐oxic atmosphere around 2.4 Ga.

## 1 INTRODUCTION

Understanding how Earth's atmosphere became O_2_‐rich is among the most important unanswered questions about deep time. The answer may inform the search for life elsewhere (Meadows et al., 2018) and elucidate the evolution of complex life on Earth (Catling et al., 2005; Catling & Zahnle, 2020; Mills et al., 2014; Reinhard et al., 2016; Sperling et al., 2013).

Compelling evidence from isotopic and inorganic redox proxies indicates atmospheric oxygen levels <10^–6^ times present in the Archean (Farquhar et al., 2000; Pavlov & Kasting, 2002; Zahnle et al., 2006), low to intermediate oxygen levels in the Proterozoic (Lyons et al., 2014; Planavsky et al., 2014, 2018; Zhang et al., 2016), and a multistep transition starting in the Neoproterozoic and reaching near‐modern pO_2_ by the late Paleozoic (Dahl et al., 2010; Krause et al., 2018; Shields‐Zhou & Och, 2011). Explanations for Earth's atmospheric oxygen accumulation can be broadly divided into increases in oxygen sources or decreases in oxygen sinks: either oxygen sources increased over Earth history or efficient oxygen sinks declined to trigger transitions between anoxic, then low oxygen, and finally near‐modern levels of atmospheric oxygen (Catling & Claire, 2005; Catling & Kasting, 2017, Ch. 10; Holland, 2002).

At any point in time, the dominant source of atmospheric oxygen is organic carbon burial because burying reduced carbon leaves behind the oxygen generated by photosynthesis (similarly, the burial of sulfides may have also contributed to net oxidation [e.g., Berner & Canfield, 1989; Holland, 2002]). Since life preferentially incorporates isotopically light carbon during carbon fixation, by assuming mass balance between inputs and outputs on long timescales, carbon isotopes can be interpreted to infer the organic burial relative to total carbon burial,$f_{\text{org}}$ (Broecker, 1970; Garrels & Perry, 1974; Hayes et al., 1999; Schidlowski, 1988; Wickman, 1956):

(1) $$f_{\text{org}}=\frac{\delta^{13}C_{\text{Burial\_carb}}‐\delta^{13}C_{\text{inputs}}}{\delta^{13}C_{\text{Burial\_carb}}‐\delta^{13}C_{\text{Burial\_org}}}$$

here, $\delta^{13}C_{\text{inputs}}$ (‰) is the carbon isotopic value of all carbon inputs into the atmosphere–ocean system, $\delta^{13}C_{\text{Burial\_carb}}$ (‰) is the isotopic value of buried carbonates, and $\delta^{13}C_{\text{Burial\_org}}$ (‰) is the isotopic value of buried organic matter.

If anoxygenic photosynthesis, or even chemosynthesis, was the dominant source of Archean organic matter, then the burial of organic carbon does not add molecular oxygen to that atmosphere directly. However, carbon fixation still produces an oxidized product (e.g., Fe^3+^ in the case of iron‐oxidizing phototrophs or sulfate in the case of H_2_S oxidation). Consequently, organic matter burial still contributes to surface oxidation prior to the advent of oxygenic photosynthesis, assuming that the oxidized product (e.g., Fe^3+^) is not subducted at a greater rate than redox‐equivalent organic carbon.

The mean value of carbon isotopes in sedimentary marine carbonates and organics is remarkably constant over Earth history (see Section 3). This has been interpreted as reflecting a relatively invariant oxygen source flux from organic carbon burial relative to carbon inputs, implying that increases in atmospheric oxygen required decreases in oxygen sinks (Holland, 2002, 2009; Kump et al., 2009; Rothman, 2015). A statistical analysis of the carbon isotope record with the simple, standard mass balance of Equation 1 and fixed $\delta^{13}C_{\text{inputs}}$ revealed that modest changes in fractional organic burial—a factor of 1.2–2.0 over Earth history with 95% confidence—are consistent with the carbon isotope record, but these changes are probably not enough to explain the anoxic Archean atmosphere (Krissansen‐Totton et al., 2015). An anoxic Archean atmosphere requires that the predicted oxygen source from organic burial be overwhelmed by fast and efficient oxygen sinks, primarily reducing, outgassed volatiles (see Methods). Various hypotheses for explaining Earth's secular oxidation with declining O_2_ sinks have been proposed (e.g., Catling et al., 2001; Claire et al., 2006; Gaillard et al., 2011; Holland, 2009; Kasting et al., 1993; Kump & Barley, 2007).

However, approximately constant fractional organic burial over Earth history creates problems. Total carbon burial in the Archean was likely comparable to or larger than modern burial on account of elevated outgassing rates and higher weathering fluxes (Avice et al., 2017; Kipp et al., 2021; Krissansen‐Totton et al., 2018). Constant organic burial fraction therefore implies absolute Archean organic burial comparable to or greater than modern. While high Precambrian phosphorus levels have been proposed (Konhauser et al. 2007; Planavsky et al. 2010), limited availability of reductants such as H_2_, Fe^2+^, and H_2_S and evidence for low marine phosphorus levels suggest primary productivity was probably small on the Precambrian Earth (Canfield et al., 2006; Kipp & Stüeken, 2017; Laakso & Schrag, 2014; Reinhard et al., 2017; Ward et al., 2019). Moreover, even allowing for higher Precambrian O_2_ sinks, large absolute organic burial fluxes tend to predict an oxic atmosphere (Laakso & Schrag, 2018), contrary to proxy evidence, although self‐consistent model scenarios with high Archean organic burial, high reductant input, and an anoxic atmosphere have been proposed, albeit without exploring consistency with the $\delta^{13}C$ record (Alcott et al., 2019; Kipp et al., 2021).

One possible solution to these problems is that the uniform carbon isotope record is decoupled from fractional organic burial. Ideas proposed to explain this potential decoupling can be broadly divided into (a) missing sink hypotheses and (b) varying $\delta^{13}C_{\text{inputs}}$ hypotheses. Missing sink hypotheses include the possibility that carbonate precipitation in the seafloor is isotopically distinct from shelf carbonates and is not properly accounted for in standard isotope mass balance calculations (Bjerrum & Canfield, 2004), or that isotopically light authigenic carbonates have been neglected (Schrag et al., 2013).

An alternative explanation for decoupling the carbon isotope record from organic burial is varying $\delta^{13}C$ of carbon inputs (Equation 1). Estimates of fractional organic burial through time typically assume that $\delta^{13}C_{\text{inputs}}$ equals the mantle value of −5 ± 1‰ (Deines & Gold, 1973; Des Marais & Moore, 1984; Shirey et al., 2013) throughout Earth history. In practice, the isotopic composition of inputs into the atmosphere–ocean system is the combination of mantle outgassing, metamorphic degassing and contributions from the weathering of carbonates and organic matter, the latter of which includes photochemical oxidation of thermogenic methane.

Changes in oxidative weathering fluxes could result in carbon inputs that differ from mantle values over time. Bekker and Holland (2012) argued that the GOE may have marked a transition from a low oxidative weathering regime where virtually all sedimentary organic carbon was mechanically recycled to a *p*O_2_‐buffered regime where oxidative weathering fluxes depend positively on atmospheric oxygen. Derry (2014) argued that under reducing conditions, incomplete oxidative weathering of organic matter, which is ^13^C‐depleted, would have resulted in isotopically heavier C inputs into the atmosphere–ocean system because the net weathering flux would be biased toward carbonates, which are ^13^C‐enriched relative to the mantle. In that case, the calculation of $f_{\text{org}}$ assuming a mantle‐like $\delta^{13}C_{\text{inputs}}$ value (Equation 1) would over‐estimate true Precambrian fractional organic burial.

Daines et al. (2017) further quantified this effect by incorporating O_2_‐dependent organic burial using the oxidative weathering model of Bolton et al. (2006) into a carbon–oxygen cycle model. They showed that changes in organic burial may not be reflected in the carbon isotope record because of the oxygen dependence of carbon inputs leading to changes in $\delta^{13}C_{\text{inputs}}$. Oxygen‐dependent organic weathering inputs were also incorporated into biogeochemical cycle modeling to help explain large excursions in the carbon isotope record without invoking extreme redox imbalances (Miyazaki et al., 2018).

These studies establish that fractional organic burial may not track the carbon isotope record in a straightforward way due to variations in $\delta^{13}C_{\text{inputs}}$. Moreover, it is challenging to independently constrain $\delta^{13}C_{\text{inputs}}$ because of its degeneracy with $f_{\text{org}}$ (Equation 1), although statistical arguments have been presented that loosely constrain $\delta^{13}C_{\text{inputs}}$ based on correlations between carbonate $\delta^{13}C_{\text{Burial\_carb}}$ and $\delta^{13}C_{\text{Burial\_carb}}‐\delta^{13}C_{\text{Burial\_org}}$ (Derry, 2010; Rothman et al., 2003). The possible implications of changing $\delta^{13}C_{\text{inputs}}$ for Earth's organic burial history over 4 Ga have not been quantified.

Here, we explore the “variable $\delta^{13}C_{\text{inputs}}$ hypothesis” more generally by using a self‐consistent carbon cycle model to estimate the extent to which the carbon isotope record allows for changes in organic burial over Earth history. Crucially, on rock cycle timescales (i.e., hundreds of Myr), $\delta^{13}C_{\text{inputs}}$ is not a free parameter that is independent of burial fluxes. If the carbon isotope value of inputs was to greatly diverge from mantle values, then this would eventually propagate to yield secular changes in the $\delta^{13}C$ of buried carbonates and organics larger than those observed. The carbon isotope values of crustal and mantle reservoirs are therefore tracked in our model.

We conduct an inverse analysis where the carbon isotope record, along with proxy constraints on fluxes, reservoirs, and redox conditions, are used to constrain a self‐consistent model of Earth's carbon cycle evolution. We also evaluate whether the carbon isotope record is consistent with sufficiently large changes in fractional organic burial over Earth history to explain the rise of oxygen without recourse to declining O_2_ sinks.

## 2 METHODS

### 2.1 Forward model

**FIGURE 1:**
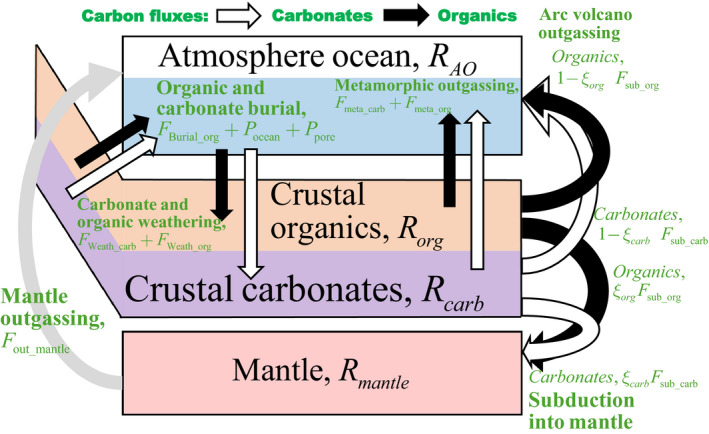
Schematic diagram of our carbon cycle model. White arrows show fluxes of carbonate carbon, black arrows show fluxes of organic carbon, and the gray arrow shows mantle outgassing of carbon. Green text denotes fluxes, and black text denotes reservoirs. Fluxes are as follows: organic burial, *F*_Burial_org_; carbonate weathering, *F*_Weath_carb_; organic oxidative weathering, *F*_Weath_org_; carbon outgassing from metamorphic carbonates and organics, *F*_meta_carb_ and *F*_meta_org_, respectively; mantle carbon outgassing, *F*_out_mantle_; and fractions, $\xi_{\text{carb}}$ and $\xi_{\text{org}}$, of subducted carbonates and organics, *F*_sub_carb_ and *F*_sub_org_, respectively. Carbon reservoirs are self‐explanatory. Associated alkalinity fluxes are not shown

Our model of the carbon cycle evolution is a modified version of the carbonate–silicate cycle model of Krissansen‐Totton et al. (2018) and Krissansen‐Totton and Catling (2017). Organic carbon burial and weathering have been added, and mantle and crustal reservoirs of carbon and isotope abundance are now tracked. A complete description of the model is provided in Text S1 and the Python code is available online upon publication. Here, we provide a description of its key components along with a schematic diagram (Figure 1).

The model tracks four reservoirs of carbon and one reservoir of carbonate alkalinity (the solid Earth is assumed to be an infinite reservoir of alkalinity):

$R_{\mathrm{AO}}$, moles of carbon in atmosphere–ocean system (excluding sediments)
$R_{\mathrm{org}}$, moles of organic carbon in crust (combined oceanic and continental, including sediments)
$R_{\mathrm{carb}}$, moles of carbonate carbon in crust (combined oceanic and continental, including sediments)
$R_{\mathrm{mantle}}$, moles of carbon in mantle
$A_{\mathrm{AO}}$, carbonate alkalinity in the atmosphere–ocean system in equivalents, that is, number of moles of carbonate and bicarbonate ions in solution multiplied by their valence. We assume this is a reasonable approximation to the conservative cation charge minus the conservative anion charges throughout Earth history. Ocean carbonate alkalinity (along with total carbon) is used to calculate pH as described in Krissansen‐Totton et al. (2018).

The impact of combining continental and seafloor reservoirs is explored in Text S5 and deemed to be minimal, for our purposes. The time evolution of these reservoirs is governed by the following system of equations:

(2) $$\begin{array}{c} \frac{dR_{\text{AO}}}{dt}=F_{\text{outg}}+F_{\text{Weath\_carb}}+F_{\text{Weath\_org}}‐P_{\text{ocean}}‐P_{\text{pore}}‐F_{\text{Burial\_org}}\\ \frac{dR_{\text{org}}}{dt}=F_{\text{Burial\_org}}‐F_{\text{sub\_org}}‐F_{\text{meta\_org}}‐F_{\text{Weath\_org}}\\ \frac{dR_{\text{carb}}}{dt}=P_{\text{ocean}}+P_{\text{pore}}‐F_{\text{Weath\_carb}}‐F_{\text{sub\_carb}}‐F_{\text{meta\_carb}}\\ \frac{dR_{\text{mantle}}}{dt}=\xi_{\text{carb}}F_{\text{sub\_carb}}+\xi_{\mathrm{org}}F_{\text{sub\_org}}‐F_{\text{out\_mantle}}\\ \frac{dA_{\text{AO}}}{dt}=2F_{\text{sil}}+2F_{\text{Weath\_carb}}+2F_{\text{dis}}‐2P_{\text{ocean}}‐2P_{\text{precip}} \end{array}$$

here, $F_{\text{Weath\_carb}}$ (mol C/year) is the weathering of crustal carbonates, $F_{\text{Weath\_org}}$ (mol C/year) is the weathering of crustal organic carbon, $P_{\text{ocean}}$ (mol C/year) is the burial of carbonates in the ocean, $P_{\text{pore}}$ (mol C/year) is the precipitation of carbonates in the seafloor, $F_{\text{Burial\_org}}$ (mol C/year) is the burial of organic carbon, $F_{\text{sil}}$ (mol C/year) is the silicate weathering flux on land, and $F_{\text{dis}}$ is the dissolution of seafloor basalt (mol C/year). Terms $F_{\text{sub\_org}}$, $F_{\text{sub\_carb}}$, $F_{\text{meta\_org}}$, and $F_{\text{meta\_carb}}$ (mol C/year) refer to the subduction and metamorphic outgassing of organic and carbonate carbon, respectively. Total outgassing, $F_{\text{outg}}$ (mol C/year), is the sum of the mantle component, $F_{\text{out\_mantle}}$, metamorphic components, and subduction components:

(3) $$F_{\text{outg}}=F_{\text{out\_mantle}}+\left(F_{\text{meta\_org}}+F_{\text{meta\_carb}}\right)+F_{\text{sub\_org}}(1‐\xi_{\text{org}})+F_{\text{sub\_carb}}(1‐\xi_{\text{carb}})$$

here $\xi_{\text{org}}$ and $\xi_{\text{carb}}$ are efficiency factors describing the fractions of subducted organic and carbonate carbon, respectively, that are returned to the mantle. The remainder, $1‐\xi_{\text{org}}$ and $1‐\xi_{\text{carb}}$, is returned to the atmosphere–ocean system via arc volcanism. Detailed parameterizations and parameter ranges for all these terms are described in the supplementary material. Here, we focus on explaining the parameterizations most important for organic burial and the carbon isotope record.

### 2.2 Key parameterizations

#### 2.2.1 Organic carbon burial and weathering parameterizations

The purpose of our model is to determine the organic burial histories that are consistent with the carbon isotope record. Organic burial is determined by an interplay between biological productivity—which in turn depends on evolutionary innovation and nutrient or free energy limitations—and burial efficiency, which is a function of local redox, biological, and sedimentological factors. Rather than explicitly model this complexity, in our model, fractional organic burial fraction (*f*_org_) is a free variable that is fit to data via inverse calculations.

To study changes over Earth history, we adopt two different parameterizations for fractional organic burial and attempt to fit both to the data. This helps ensure that our conclusions are independent of the functional form chosen to represent *f*_org_ over time. First, we divide the carbon isotope record into Hadean, Archean, Proterozoic, and post‐0.5 Ga intervals. We then use three free variables, *j*_1_, *j*_2_, and *j*_3_, to represent changes in average organic burial over time. Here, $f_{\text{org}}\left(\text{Archean}\right)=j_{1}$ is the fractional organic burial in the Archean, which is then combined with a multiplicative factor *j*_2_ to give Proterozoic fractional organic burial, $f_{\text{org}}\left(\text{Proterozoic}\right)=j_{1}j_{2}$. In turn, Proterozoic organic burial is multiplied by the factor *j*_3_ to give Phanerozoic fractional organic burial, $f_{\text{org}}\left(\text{modern}\right)=j_{1}j_{2}j_{3}$. Thus, fractional organic burial is computed as follows:

(4) $$f_{\text{org}}=\left\{\begin{array}{cc} 0, & t>4.0\;\text{Ga}\\ j_{1}, & 2.5<t<4\;\text{Ga}\\ j_{1}j_{2}, & 0.5<t<2.5\;\text{Ga}\\ j_{1}j_{2}j_{3}, & t<0.5\;\text{Ga} \end{array}\right)$$

Later, we repeat our analyses using a simple linear trend for fractional organic burial. This approach of using a several different functions to fit carbon isotope data is analogous to that adopted in Krissansen‐Totton et al. (2015).

The absolute organic burial flux (mol C/year) is calculated, as follows, by assuming that the atmosphere–ocean system is in steady state on long timescales:

(5) $$F_{\text{Burial\_org}}=f_{\text{org}}\times \left(\text{total}\;\text{carbon}\;\text{inputs}\right)=f_{\text{org}}\times \left(F_{\text{Weath\_carb}}+F_{\text{Weath\_org}}+F_{\text{outg}}\right)$$

The relative contributions of organic weathering, carbonate weathering, and outgassing control the average isotopic composition of carbon inputs, $\delta^{13}C_{\text{inputs}}$, which is used for calculating fractional organic burial through time (Equation 1). Outgassing fluxes are controlled by the combination of mantle outgassing, metamorphic outgassing, and arc volcanism, which in turn depend on some combination of internal heatflow, crustal recycling rates, and crustal carbon reservoirs. We assume a broad range for these parameters consistent with literature estimates (Text S1).

Organic weathering is assumed to be a combination of oxidative weathering (first term) and photochemical oxidation of thermogenic methane (second term):

(6) $$F_{\text{Weath\_org}}=F_{\text{oxid}}^{\text{modern}}\frac{R_{\text{org}}}{R_{\mathrm{org}}^{\text{modern}}}\frac{f_{\text{land}}}{f_{\text{land}}^{\text{modern}}}{\left(\frac{{\text{pO}}_{2}}{{\text{pO}}_{2}^{\text{modern}}}\right)}^{0.3}+F_{\text{thermo}}^{\text{modern}}\frac{R_{\text{org}}}{R_{\text{org}}^{\text{modern}}}$$

Here, atmospheric oxygen relative to modern, ${\text{pO}}_{2}/{\text{pO}}_{2}^{\text{modern}}$, is 10^–9^ in the Archean, 1.0 in the Phanerozoic, and an unknown parameter in the Proterozoic with a range from 10^–3^ to 10^–1^ PAL (see Table 1 and discussion of priors in Section 2.6). The fraction $f_{\text{land}}/f_{\text{land}}^{\text{modern}}$ describes the area of subaerial land relative to modern which evolves with time according to the parameterization described in Krissansen‐Totton et al. (2018). This parameterization allows for Archean subaerial land fractions from 0% (negligible subaerial land) to 50% modern, and smoothly transitions to 100% modern around 2–3 Ga. The dependence of our results on land fraction is explored in sensitivity tests. Both oxidative weathering and thermogenic methane are assumed to be proportional to the crustal organic reservoir relative to modern, $R_{\text{org}}/R_{\text{org}}^{\text{modern}}$.

**TABLE 1:** Data fitted by inverse analysis

Model variable	Observed value	Uncertainty in observed value (1σ)	References
Carbonate isotope record, $\delta^{13}C_{\text{carb}}$	Time series from 3.8 Ga to 0 Ga (‰)	Standard deviation of 200 Myr bins	Filtered carbon isotope data from Krissansen‐Totton et al. (2015)
Organic carbon isotope record, $\delta^{13}C_{\text{org}}$	Time series from 3.8 Ga to 0 Ga (‰)	Standard deviation of 200 Myr bins	Filtered carbon isotope data from Krissansen‐Totton et al. (2015)
Mantle outgassing at 3.3 Ga relative to modern, $F_{\text{out\_mantle}}/F_{\text{out\_mantle}}^{\text{modern}}$	8.1	3.9	Avice et al. (2017)
Modern, pre‐industrial atmospheric carbon dioxide_,_ ${\text{pCO}}_{2}^{\mathrm{modern}}$	280 ppm	0.2 log_10_ unit	Pearman et al. (1986). Error encompasses pCO_2_ variability in last 3 Myrs (Willeit et al., 2019)
Modern surface temperature,$T_{S}^{\;\mathrm{mod}\;}$	285.0 K	5.0 K	Climate model returns *T*_S_ = 285 K at 280 ppm CO_2_ and modern luminosity (Krissansen‐Totton et al., 2018). Uncertainty is approximate glacial‐interglacial variance
Modern ocean pH	8.2	0.5	Halevy and Bachan (2017), Pilson (2012, p. 127)
Modern mantle carbon reservoir, $R_{\text{mantle}}^{\text{modern}}$	2 × 10^22^ mol C	10^22^ mol C	Coltice et al. (2004), Dasgupta and Hirschmann (2010), Javoy et al. (1982), Sleep and Zahnle (2001)
Modern crustal carbonate reservoir, $R_{\text{carb}}^{\text{modern}}$	9.4 × 10^21^ mol C	5.5 × 10^21^ mol C	Gao et al. (1998), Hartmann et al. (2012), Wedepohl (1995)
Modern crustal organic carbon reservoir, $R_{\text{org}}^{\text{modern}}$	1.66 × 10^21^ mol C	0.55 × 10^21^ mol C	10%–20% of total crustal carbon reservoir (Gao et al., 1998; Hartmann et al., 2012; Wedepohl, 1995)
Archean oxidation parameter, Koxy	<1.0	0.20^a^	Catling and Kasting (2017)
Archean mantle $\delta^{13}C$	−5.5‰	3.0	Shirey et al. (2013)
Modern mantle $\delta^{13}C$	−5.5‰	0.5	Deines and Gold (1973), Des Marais and Moore (1984)
Organic Burial: Weathering ratio, $F_{\text{Burial\_org}}/F_{\text{Weath\_org}}$	>1.0	N/A	Required for long‐term oxygen accumulation in surface reservoirs

Note

The second column shows their assumed observed value, and the third column shows observational uncertainty.

^a^ Given median values for organic burial and outgassing in the Archean, varying the sulfide and Fe(II) burial term in the expression for Koxy from 0 (none in Archean) to 100% (unchanged since the Archean) results in variations in Koxy of about 0.2.

Modeling the oxygen dependence of organic weathering is non‐trivial. In general, organic burial rates will depend on boundary layer kinetics and local uplift rates: If uplift rates are slow, then most organic carbon will be oxidized under oxic conditions regardless of the precise atmospheric O_2_ abundance; if uplift rates are rapid, then oxidative kinetics become important and organic weathering will vary with atmospheric O_2_ (Bolton et al., 2006). Rather than explicitly model the boundary layer and attempt to integrate global uplift rates, we instead assume that the global O_2_ dependence of oxidative weathering can be approximated with a ${\text{RO}}_{2}^{0\text{.3}}$ power law (Bolton et al., 2006; Daines et al., 2017), where RO_2_ is the ratio of oxygen level relative to modern, ${\text{pO}}_{2}/{\text{pO}}_{2}^{\text{modern}}$. However, we also explore other endmember cases such as ${\text{RO}}_{2}^{0\text{.5}}$ (Chang & Berner, 1999) and a negligible O_2_ dependence, where the latter represents a scenario in which all oxidative weathering is erosion‐limited and depends on organic content of crust.

In addition to the O_2_‐dependent organic weathering term, we also include an O_2_‐independent organic weathering component to represent thermogenic methane oxidation. Most of the carbon in thermogenic methane is ultimately photochemically oxidized regardless of atmospheric oxygen content—and regardless of whether its hydrogen escapes—thereby contributing to the oxidation of reduced carbon in the crust. The relative contributions of oxidative weathering ($F_{\text{oxid}}^{\text{modern}}$) and thermogenic methane ($F_{\text{thermo}}^{\text{modern}}$) to the modern organic weathering flux are not well constrained, and so, a broad range for each is assumed (see discussion below). By assuming a broad range for each of these modern fluxes and mixing the O_2_‐dependent and O_2_‐independent terms (Equation 6), we are effectively allowing for a very broad range of oxygen dependencies for overall organic weathering. We also investigated the sensitivity of our results to changing the parameter ranges in Equation 6.

#### 2.2.2 Carbonate weathering parameterization

Carbonate weathering is parameterized as follows:

(7) $$F_{\text{Weath\_carb}}=F_{\text{Weath\_carb}}^{\text{modern}}\left(\frac{R_{\text{carb}}}{R_{\text{carb}}^{\text{modern}}}\right)\left(\frac{f_{\text{land}}}{f_{\text{land}}^{\text{modern}}}\right){\left(\frac{{\text{pCO}}_{2}}{{\text{pCO}}_{2}^{\text{modern}}}\right)}^{\alpha }\exp\left(\frac{\Delta T_{S}}{T_{e}}\right)$$

here, $R_{\text{carb}}/R_{\text{carb}}^{\text{modern}}$ is the crustal carbonate carbon reservoir relative to modern, and $F_{\text{Weath\_carb}}^{\text{modern}}$ (mol C/year) is the modern carbonate weathering flux, which is an unknown parameter ranging from 7 to 25 Tmol C/year (Table 2). The difference between mean surface temperature and modern mean surface temperature is specified by $\Delta T_{S}$ (K), and the e‐folding temperature, *T*_e_ (K), controls the temperature dependence of weathering. Similarly, the exponent, *α*, controls the dependence of carbonate weathering on relative atmospheric carbon dioxide, ${\text{pCO}}_{2}/{\text{pCO}}_{2}^{\text{modern}}$. Surface temperature is calculated using the climate model described in Krissansen‐Totton et al. (2018).

**TABLE 2:** Unknown parameters in inverse model with uniform prior ranges

Unknown model parameter	Uniform prior range	Reference/justification
Archean fractional organic burial, *j*_1_	0.01–0.5	Broad enough to accommodate both large increases or decreases over Earth history
Proterozoic fractional organic increase, *j*_2_	0.5–5.0	Broad enough to accommodate large increases or decreases over Earth history
Phanerozoic fractional organic burial increase, *j*_3_	0.5–5.0	Broad enough to accommodate large increases or decreases over Earth history
Modern organic weathering flux via oxidative weathering, $F_{\text{oxid}}^{\text{modern}}$	2 × 10^12^–5 × 10^12^ mol C/year	Lasaga et al. (1985), Lenton et al. (2018), Petsch (2014). See discussion in main text
Modern organic weathering flux via photochemical oxidation of thermogenic methane, $F_{\text{thermo}}^{\text{modern}}$	1 × 10^12^–4 × 10^12^ mol C/year	Etiope et al. (2019), Saunois et al. (2020)
Proterozoic oxygen relative to modern, $\log_{10}\left(\frac{{\text{pO}}_{2}^{\text{Proterozoic}}}{{\text{pO}}_{2}^{\text{modern}}}\right)$	−3 to −1	Lyons et al. (2014), Planavsky et al. (2018). Oxygen not photochemically stable below ~10^−3^ PAL (Zahnle et al., 2006)
Initial (late Hadean) CO_2_, $\log_{10}\left({\text{pCO}}_{2}^{\text{init}}\right)$ (bar)	−2.5 to 1.5	Krissansen‐Totton et al. (2018)
Direct CO_2_ dependence of continental weathering, α	0.1 to 0.5	Krissansen‐Totton et al. (2018)
Direct temperature dependence of continental weathering, *T*_e_	10 to 40 K	Krissansen‐Totton and Catling (2017)
Exponent controlling dependence of outgassing on heatflow, *µ*	0 to 2	Modified from Krissansen‐Totton et al. (2018) to allow for broader outgassing histories
Exponent controlling dependence of spreading rate on heatflow, β	1 to 2	Krissansen‐Totton et al. (2018)
Exponent controlling heatflow evolution, *n*_out_	0 to 2	Krissansen‐Totton et al. (2018)
Direct temperature dependence of seafloor weathering, *E*_bas_	60 to 100 kJ/mol	Krissansen‐Totton and Catling (2017)
Modern Earth carbonate weathering flux, $F_{\text{Weath\_carb}}^{\text{modern}}$	7 × 10^12^ to 25 × 10^12^ mol C/year	Berner and Mackenzie (2011), Gaillardet et al. (1999), Hartmann et al. (2009, their table 2), Milliman (1993). See discussion in main text
Archean land fraction relative to modern, $f_{\text{land}}^{\text{Archean}}/f_{\text{land}}^{\text{modern}}$	0.0 to 0.5	Krissansen‐Totton et al. (2018)
Timing of subaerial continent growth, $t_{\text{grow}}$	2.0 to 3.0 Ga	Krissansen‐Totton et al. (2018)
Initial (late Hadean) pH	5.5 to 8.0	Halevy and Bachan (2017), Krissansen‐Totton et al. (2018)
Initial (late Hadean) crustal carbonate reservoir, $\log_{10}\left(R_{\text{carb}}^{\text{init}}\right)$, (mol C)	10^18^ to 10^22^ mol C	Broad range because unknown whether carbon resided in Hadean mantle or crust
Initial (late Hadean) mantle carbon reservoir, $R_{\text{mantle}}^{\text{init}}$ (mol C)	10^21^ to 4 × 10^22^ mol C	Total carbon conserved over Earth history, but uncertainty in modern mantle reservoir (Coltice et al., 2004; Dasgupta & Hirschmann, 2010; Javoy et al., 1982; Sleep & Zahnle, 2001)
Organic weathering subduction efficiency^a^, $\xi_{\text{org}}$.	0.2–0.8	Dasgupta and Hirschmann (2010), Duncan and Dasgupta (2014), Plank and Manning (2019)
Modern carbonate weathering subduction efficiency^a^,$\xi_{\text{carb}}\left(\text{modern}\right)$	0.2–0.8	See Text S4 for how $\xi_{\text{carb}}$ evolves as the mantle cools

Note

These variables may be constrained by fitting the model to the data in Table 1.

^a^ We apply the additional constraint that $\xi_{\text{org}}>\xi_{\text{carb}}\left(\text{modern}\right)$.

There is uncertainty in the global temperature dependence and CO_2_ dependence of carbonate weathering. Increasing CO_2_ in isolation causes an increase in carbonate dissolution, but increasing air temperature may lower carbonate dissolution due to changes in the calcite equilibrium state (Gaillardet et al., 2018; Romero‐Mujalli et al., 2018). In contrast, a positive relationship between temperature and carbonate weathering is typically assumed in global models attributable to a positive correlation between global runoff and weathering (Le Hir et al., 2008; Shields & Mills, 2017). We sample a broad range of temperature and CO_2_ dependencies to accommodate scenarios ranging from essentially no dependence of weathering on these variables to a strong positive relationship (Table 2). As a sensitivity test, we also consider a Michaelis–Menten dependence for carbonate weathering dependence on CO_2_ to capture decoupling between soil pCO_2_ and atmospheric pCO_2_.

#### 2.2.3 Subduction efficiency parameterization

Estimates of subduction efficiency for the modern Earth are variable, ranging from ~20% to 80% (Dasgupta & Hirschmann, 2010; Duncan & Dasgupta, 2014; Plank & Manning, 2019). Additionally, a hotter Archean mantle may have more efficiently devolatilized downgoing carbon (Hayes & Waldbauer, 2006). Specifically, subducted carbonates are volatilized more readily than organic carbon under hotter mantle conditions (Duncan & Dasgupta, 2017). Changing subduction efficiencies may have implications for interpreting the isotope record because preferential subduction of organics may produce crustal carbonates that are isotopically heavier in the Archean. Consequently, inputs into the atmosphere–ocean system—the weighted sum of weathering and outgassing carbon inputs—may have been more ^13^C enriched in the past.

To investigate the possible effect of this preferential subduction on the carbon isotope record and implied organic burial history, we allowed the efficiency of carbonate subduction to the mantle, $\xi_{\text{carb}}$, to scale with internal heatflow, evolving toward a highly uncertain modern value (20%–80%). This approach is based on the slab decarbonation parameterization adopted by Höning et al. (2019) and is described in Text S4. We assume organic carbon has subducted with constant efficiency, $\xi_{\text{org}}$, over Earth history sampled from 20% to 80% (Table 2). Additionally, we constrain organic carbon subduction to always be more efficient than carbonate subduction (Duncan & Dasgupta, 2017). Sensitivity tests are conducted with constant subduction efficiency for both carbonate and organic carbonate to isolate the influence of evolving subduction efficiencies on the carbon isotope record.

### 2.3 Forward model: carbon isotopes

In addition to tracking the time evolution of carbon reservoirs, the model also calculates the carbon isotope evolution of these reservoirs. The full system of equations is described in Text S2. Both total carbon and ^13^C carbon are conserved over Earth history in all calculations (see Text S5). Following Schidlowski (2001), carbon isotope fractionation is introduced by imposing an average 28‰ fractionation between buried organic carbon and buried carbonates based on the approximately constant observed mean difference between $\delta^{13}C_{\text{Burial\_org}}$ and $\delta^{13}C_{\text{Burial\_carb}}$ over Earth history (Krissansen‐Totton et al., 2015).

### 2.4 Inverse model

The carbon cycle model described above was used as the forward model in an inverse analysis designed to constrain the magnitude of the change in fractional organic burial over Earth history (i.e., solve for *j*_1_, *j*_2_, and *j*_3_). The inverse analysis was implemented using the “emcee” package in Python (Foreman‐Mackey et al., 2013) and posterior distribution figures were created using the “corner” module in Python (Foreman‐Mackey, 2016). The emcee package implements an affine‐invariant Markov Chain Monte Carlo (MCMC) ensemble sampler. Many walkers in the MCMC algorithm explore parameter space, evaluating the likelihood function for each model call. The likelihood is determined by the difference between model outputs (MOD) and geochemical and geophysical constraints (OBS):

(8) $$\log\left(L\right)=‐\frac{1}{2}\left(\sum_{k}\sum_{i}\frac{{\left({\text{OBS}}_{i,k}‐{\text{MOD}}_{i,k}\right)}^{2}}{\sigma_{i,k}^{2}}\right)‐\frac{1}{2}\sum_{k}\sum_{i}\log\left(2\pi \sigma_{i,k}^{2}\right)$$

here, ${\text{OBS}}_{i,k}$, ${\text{MOD}}_{i,k}$, and $\sigma_{i,k}$ are the observed (proxy) value, model value, and uncertainty in the observed value of the *i*th data point of the *k*th variable, respectively. The *k* summation is over the thirteen variables for which we have observational constraints described below. For example, suppose in a forward model run the modern surface temperature is 289 K. In that case, ${\text{MOD}}_{1,k}$ = 289 K, whereas the observed value with uncertainty is ${\text{OBS}}_{1,k}$ = $T_{S}^{\;\mathrm{mod}\;}$= 285 K, and $\sigma_{1,k}$ = 5 K (Table 1). There is no summation over *i* for surface temperature because only one modern value is used to constrain the model. However, for the carbon isotope record contributions to the likelihood function, *i* would denote the summation over 200 Myr binned carbon isotope data.

We used 500 walkers and 10,000 model steps—that is a total of 5 million forward model calls—to build posterior distributions for our parameters. The initial walker positions were randomized, and a 2,000 step burn‐in was discarded.

### 2.5 Observational constraints

Table 1 summarizes the data used to constrain the inverse analysis. Carbonate and organic carbon isotope time series were obtained from Krissansen‐Totton et al. (2015). Archean mantle outgassing constraints were obtained from Xe isotopes (Avice et al., 2017). Modern reservoir values were taken from literature compilations.

Atmospheric redox state is calculated using the $K_{oxy}$ parameter (Claire et al., 2006; Krissansen‐Totton et al., 2015):

(9) $$K_{oxy}=\frac{F_{\text{Burial\_org}}+F_{\text{Burial\_other}}\times \left(f_{\mathrm{org}}/f_{\mathrm{org}}^{\mathrm{modern}}\right)}{F_{\text{reduced}}\times \left(F_{\text{outg}}/F_{\text{outg}}^{\mathrm{modern}}\right)}$$

The $K_{oxy}$ parameter is the ratio between the oxygen source flux ($F_{\text{Burial}\_\text{org}}$ and oxygen produced from the burial of other reduced species) and fast and efficient oxygen sinks ($F_{\text{reduced}}$, scaled by a ratio of past outgassing to modern), here assumed to be dominated by oxidizable volcanic and metamorphic gases (H_2_, CO, etc.). When there is a larger flux of oxidizable gases than O_2_ to oxidize them, $K_{oxy}$ < 1, meaning that fast sinks dominate and atmospheric chemistry dictates that the atmosphere is anoxic. Reductants that exceed what is required to reduce O_2_ sourced from organic carbon burial will accumulate in the atmosphere. In contrast, when $K_{oxy}$ > 1, O_2_ will accumulate until balanced by continental oxidative weathering of sulfides and organics (Claire et al., 2006; Kasting, 2013).

We have adapted $K_{oxy}$ to apply to our specific model. Here, the second term in the numerator represents the oxygen source from sulfide and Fe(II) burial, which is crudely assumed to scale with organic burial (Krissansen‐Totton et al., 2015), although uncertainties in other burial fluxes are considered in our calculations such that Archean $F_{\text{Burial\_other}}$ values between zero and modern are permitted (see Table 2). We adopt a modern value for oxygen produced by sulfide and Fe(II) burial of $F_{\text{Burial\_other}}$ = 5.2 Tmol O_2_/year (Holland, 2002; Krissansen‐Totton et al., 2015). The term in the denominator is the outgassing flux of oxygen‐consuming reduced gases such as H_2_, CH_4_, and CO, which we assume to scale with total outgassing flux. In other words, a constant mantle redox state over Earth history is implicit in our nominal model to isolate the effect of secular change in O_2_ fluxes from molar equivalent organic burial fluxes. However, we do test the sensitivity to this assumption by also considering a more reduced early mantle. Additionally, modern O_2_ consumption by reduced volcanic and metamorphic gases is assumed to be $F_{\text{reduced}}$ = 2.4 Tmol O_2_/year (Holland, 2002). In our inverse analysis, we apply the constraint $K_{oxy}$ < 1.0 in the Archean. Note that $K_{oxy}$ > 1 is not completely forbidden; however, the log‐likelihood of model runs with $K_{oxy}$ > 1 is penalized with weighting specified by the uncertainty in the flux terms described above (see Table 1). We also require that organic burial always exceeds organic weathering to ensure our model redox budget is consistent with long‐term oxygen accumulation, although this condition is naturally satisfied by our weathering and burial parameterizations and so neglecting this constraint does not change our conclusions.

The model is initialized at 4.1 Ga and runs forward in time (Text S3). This is necessary because the solid Earth reservoirs are not necessarily in steady state and thus the directionality of time is needed. Modern pre‐industrial pCO_2_, ocean pH, and surface temperature are also used as observational constraints in the inverse analysis (Table 1). In other words, after 4.1 Ga initialization, the model must finish at 0 Ga with appropriate modern conditions.

### 2.6 Uncertainty in modern absolute fluxes

We opted not to use modern fractional organic burial to constrain the model because this value is not precisely known independently of carbon isotope mass balance calculations and thus is potentially a useful output from the inverse model. However, prior ranges for modern absolute flux must be specified. We adopted a prior range for modern oxidative weathering, $F_{\text{oxid}}^{\text{modern}}$, of 2–5 Tmol C/year (Lasaga et al., 1985; Lenton et al., 2018; Petsch, 2014). In combination with 1–4 Tmol C/year thermogenic methane oxidation, $F_{\text{thermo}}^{\text{modern}}$ (Etiope et al., 2019; Saunois et al., 2020), this implies a 3–9 Tmol C/year range for modern organic weathering. Higher oxidative weathering estimates exist in the literature (e.g., Wallmann & Aloisi, 2012), but these are disfavored because the maximum amount of organic material delivered to the ocean via erosion is ~8 Tmol/year (Daines et al., 2017; Milliman & Syvitski, 1992), and organic matter oxidation is known to be incomplete even under the modern oxic atmosphere (e.g., Galy et al., 2008; Hilton et al., 2011; Petsch, 2014). We explore sensitivity tests allowing for higher modern organic weathering fluxes (see discussion).

Literature estimates of modern carbonate weathering fluxes also vary substantially. Global integration of lithology‐dependent weathering fluxes suggests that the modern carbonate weathering flux could be as low as 7 Tmol C/year (Hartmann et al., 2009); however, the total carbonate burial flux may be as high as 30–40 Tmol C/year (Milliman, 1993), which would imply a carbonate weathering flux around 20–30 Tmol C/year, assuming a ~10 Tmol C/year contribution from silicate weathering (Gaillardet et al., 1999; Lenton et al., 2018). Measurements of sedimentation rates and cation riverine delivery suggest modern carbonate burial fluxes around 20–25 Tmol C/year (Berner & Mackenzie, 2011; Gaillardet et al., 1999), which would imply carbonate weathering fluxes around 10–15 Tmol C/year after correcting for the silicate weathering contribution. We conservatively adopt a very wide prior from 7 to 25 Tmol C/year, but also consider more narrow ranges in sensitivity tests.

### 2.7 Apparent fractional organic burial with −5.5‰ input carbon

As noted above, fractional organic carbon burial rates may not necessarily be reflected in the carbon isotope record due to varying carbon isotope inputs into the atmosphere–ocean system. To illustrate this, it is helpful to define the “apparent” fractional organic carbon burial, $f_{\text{org}(‐5.5)}$. This is the *f*_org_ value that would be calculated if carbon inputs, $\delta^{13}C_{\text{inputs}}$ in Equation 1, were assumed to always have an isotope ratio identical to that of the modern mantle, $\delta^{13}C_{\text{mantle}}^{\text{modern}}=‐5.5$‰, that is,

(10) $$f_{\text{org}(‐5.5)}=\frac{\delta^{13}C_{\text{Burial\_carb}}‐\delta^{13}C_{\text{mantle}}^{\text{modern}}}{\delta^{13}C_{\text{Burial\_carb}}‐\delta^{13}C_{\text{Burial\_org}}}$$

If organic weathering is strongly dependent on atmospheric oxygen or if preferential subduction of organics creates a secular trend in crustal inputs, then $\delta^{13}C_{\text{inputs}}\neq \delta^{13}C_{\text{mantle}}^{\text{modern}}$ and $f_{\text{org}}\neq f_{\text{org}(‐5.5)}$. Changes in absolute weathering fluxes also affect $\delta^{13}C_{\text{inputs}}$ and thus may also contribute to a decoupling of $f_{\text{org}}$ and $f_{\text{org}(‐5.5)}$. If organic weathering is instead largely independent of atmospheric oxygen and if subduction efficiencies have remained constant over Earth history, then $\delta^{13}C_{\text{inputs}}\approx \delta^{13}C_{\text{mantle}}$ and $f_{\text{org}}\approx f_{\text{org}(‐5.5)}$, as is typically assumed by most analyses of the carbon cycle (e.g., Krissansen‐Totton et al., 2015). In contrast with this apparent fractional organic burial, we refer to the actual organic burial fraction in the model as “true” *f*_org_; this is the quantity we are attempting to constrain using the isotope record.

## 3 RESULTS

**FIGURE 2:**
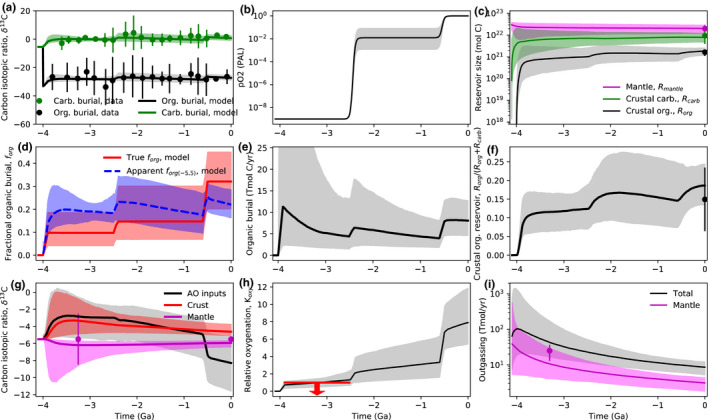
Inverse carbon cycle model outputs fit to data to constrain organic burial over Earth history. In all subplots, solid lines denote median model outputs and shaded regions denote 95% credible intervals, whereas dots with error bars are empirical constraints (Table 1). The binned carbon isotope record (every 200 Myr) for both carbonates (green dots) and organic carbon (black dots) is shown alongside the model fit (green and black shaded regions) in (a). The corresponding fractional organic burial record is shown by the red shaded region in (d) alongside the apparent fractional organic burial record (blue) that would be inferred by wrongly assuming inputs to the atmosphere–ocean system have always had mantle isotopic composition (−5.5‰). The average isotopic composition of inputs to the atmosphere–ocean system (outgassing plus weathering) is shown in (g) by the gray shaded region. The isotopic evolution of the crust (red) and mantle (magenta) is also plotted. Not the large decrease in the isotopic ratio of inputs, which is caused by the oxygen dependence of organic weathering, is coincident with the second rise of oxygen (b). Although Archean fractional organic burial was almost certainly smaller than modern (b), the absolute Archean organic burial flux (e) was comparable to, or perhaps even slightly larger than the modern flux. This is because total carbon throughput is larger in the Archean (j). The evolution of the crustal and mantle reservoirs of carbon is shown in (c) and (f), and (h) shows the atmospheric oxygenation parameter, K_oxy_ (gray shaded region), alongside the constraint that the Archean atmosphere was anoxic (red upper limit)

**FIGURE 3:**
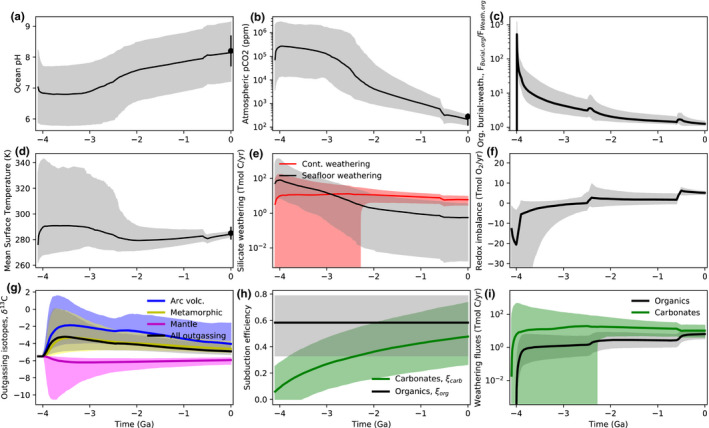
Additional inverse carbon cycle model outputs fit to data to constrain organic burial over Earth history. In all subplots, solid lines denote median model outputs and shaded regions denote 95% credible intervals, whereas dots with error bars are empirical constraints (Table 1). Subplots denote (a) surface ocean pH, (b) atmospheric CO_2_ concentration (ppmv), (c) organic burial to organic weathering ratio, (d) mean surface temperature, (e) continental silicate weathering (red) and seafloor silicate weathering fluxes (black–gray), and (f) net surface redox imbalance. Subplot (g) shows the isotopic composition of outgassing inputs, (h) shows the evolution of organic and carbonate subduction efficiencies, and (i) shows carbonate and organic weathering fluxes. Note the increase in organic weathering coincident with the Neoproterozoic rise of oxygen which drives the shift toward lighter $\delta^{13}C$ input values

**FIGURE 4:**
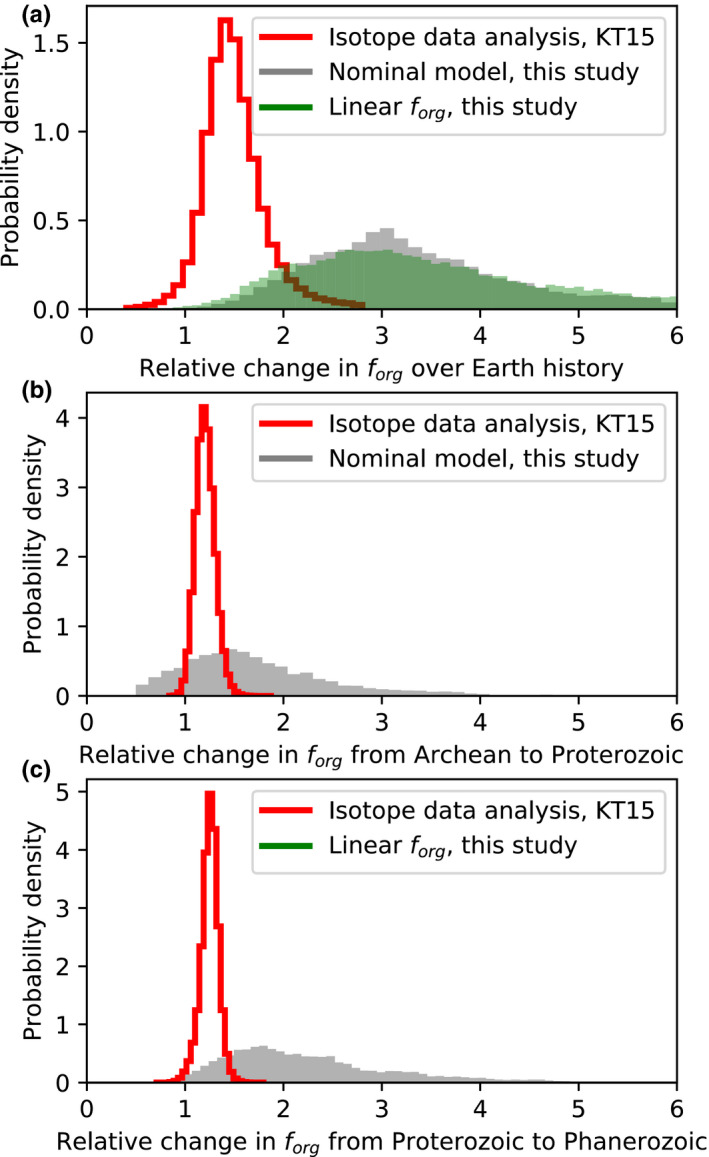
Change in fractional organic burial over Earth history from our inverse model. Comparison between organic burial changes inferred from the inverse carbon cycle analysis in this study and those from the statistical analysis in Krissansen‐Totton et al. (2015) (red), which assumed constant mantle value carbon inputs. (a) Posterior probability distribution for the relative change in fractional organic burial over Earth history according our nominal model (gray shaded). This is obtained from multiplying the Archean–Proterozoic change factor (*j*_2_) and the Proterozoic–Phanerozoic change factor (*j*_3_) from our nominal model. Also plotted is the change in fractional organic burial from 3.5 Ga to present when a linear fractional organic burial parameterization is imposed. Both parameterizations suggest that a 2–5 fold increase in fractional organic burial over Earth history can be reconciled with the carbon isotope record. The inverse analysis allows for a larger change in organic burial than in Krissansen‐Totton et al. (2015; red line) because it allows for variations in weathering input fluxes and preferential subduction of organics. (b) Shows the same comparison between this analysis (gray) and Krissansen‐Totton et al. (2015; red) except that only the Archean‐to‐Proterozoic relative change is plotted, whereas (c) shows the Proterozoic–Phanerozoic relative change in fractional organic burial

Figures 2, 3, and 4 show the results from our nominal inverse analysis. Data from Table 1 were used in the calculations to constrain carbon cycle evolution, and Table 2 shows the 22 unknown parameters in the model with their priors. Figures 2 and 3 show the time evolution of carbon cycle variables alongside constraints. Figure 4 shows the posterior probability distribution for the relative change in fractional organic burial from 4.0 Ga to present along with the increases at the Archean‐Proterozoic and Proterozoic–Phanerozoic transitions. These posterior distributions represent the spread in fractional organic burial histories permitted by the data (Table 1) according to our carbon cycle model.

Figure 2a shows the carbon isotope record binned every 200 Myr alongside the fitted model values for the crustal organic and carbonate reservoirs over time. Figure 2d shows the corresponding fractional organic burial inferred by the model. This is compared to the apparent fractional organic burial (in blue) that would be inferred if carbon isotope inputs are always assumed to be isotopically identical to the mantle (−5.5‰). These quantities diverge significantly with the Neoproterozoic rise of oxygen because the increase in oxidative weathering shifts the average isotopic composition of carbon isotope inputs toward lighter values. This can be seen clearly in Figure 2g where inputs diverge from crustal values in the Neoproterozoic. Figure 2g also shows the time evolution of the isotopic composition of the mantle. In most model runs, this does not change appreciably over Earth history due to the large size of the mantle reservoir. However, because we allow for initial conditions where Earth's carbon mostly resides in the Hadean crust (Table 2), mantle $\delta^{13}C$ may have changed early in Earth's history (as reflected in the uncertainty shading) as subducted carbon accumulated in the mantle.

Figure 2c,f show the evolution of crustal reservoirs toward their observed modern values. The relative abundance of organic carbon to total carbon in the crust (Figure 2f) is not necessarily identical to the organic burial fraction (Figure 2d) due to the preferential subduction of organics to the mantle. The median value for modern fractional organic burial is around $0.32_{‐0.07}^{+0.07}$ (1σ), whereas the modern organic carbon to total carbon crustal fraction is 0.13–0.24 (95% credible), within error or crustal inventory estimates (Table 1).

Figure 2h shows the evolution of the redox parameter K_oxy_, alongside the K_oxy_(Archean) < 1 constraint. The nominal model is within error of an anoxic Archean atmosphere. This shows that while it is possible to account for the transition from an anoxic‐to‐oxic atmosphere without recourse to declining sinks, the fit is marginal (we investigate declining outgassing sinks below). Figure 2e shows absolute organic weathering flux, and Figure 2i shows outgassing fluxes over Earth history where the Archean mantle outgassing flux is compared to that inferred from ^129^Xe (Avice et al., 2017).

Figure 3 shows other variables from the inverse analysis including the evolution of atmospheric CO_2_ (Figure 3b), surface temperature (Figure 3d), and ocean pH (Figure 3a) to their modern values. Figure 3c shows the ratio of organic burial to organic weathering. Figure 3e shows continental and seafloor silicate weathering fluxes over Earth history. There is the possibility of zero continental weathering in the Archean if subaerial land is negligible. Note that there is clear evidence of land at 3.7 Ga (Nutman et al., 2015), so this assumption is an idealized endmember case. The sensitivity of our results to continental land fraction is explored below. Similarly, carbonate weathering may be negligible in the Archean in zero land cases (Figure 3i). Figure 3g shows the isotopic composition of outgassing components. Arc volcanism is isotopically heavier than mantle outgassing by a few parts per mil, as expected due to preferential subduction of organics into the mantle (Mason et al., 2017). This is shown explicitly in Figure 3h, which plots subduction efficiencies over time for both organic and carbonate carbon. Finally, Figure 3f shows the imbalance between oxygen source and sink fluxes in the atmosphere–ocean system:

(11) $$\text{Redox}\;\text{imbalance}=F_{\text{Burial\_org}}+F_{\text{Burial\_other}}\left(f_{\text{org}}/f_{\text{org}}^{\text{modern}}\right)‐F_{\text{reduced}}\left(F_{\text{outg}}/F_{\text{outg}}^{\text{modern}}\right)‐F_{\text{Weath}\_\text{org}}$$

Since we do not attempt a full redox accounting (e.g., neglecting H escape, or detailed sulfur cycling and iron cycling), there is an imbalance between sources and sinks. However, the source–sink balance transitions from negative to positive around the GOE, which is self‐consistent.

Figure 4 shows the inferred probability distributions for the change in organic burial fraction, as constrained by our model fit to data. Figure 4a shows the relative change in fractional organic burial from the Archean to the present from our nominal model (Figure 2d). This is compared against results from a repeated inverse analysis where a different functional form is assumed for *f*_org_:

(12) $$f_{\mathrm{org}}=a_{\mathrm{grad}}\left(4.0‐t_{\mathrm{Gyr}}\right)+b_{\text{int}}$$

In this calculation, the gradient and intercept, $a_{\text{grad}}$ and $b_{\text{int}}$, are the unknown constants that replace *j*_1_, *j*_2_, and j_3_ in the nominal model, and *t*_Gyr_ is the time (in Gyr) before the present. The similarity between these two distributions in Figure 4a confirms that our assumed functional form for fractional organic burial does not affect our conclusions. Both these distributions are compared to the changes in fractional organic burial inferred from the statistical analysis in Krissansen‐Totton et al. (2015), which assumed carbon isotope inputs equal mantle values. Transitions in fractional organic burial from the Archean to Proterozoic and Proterozoic to Phanerozoic are also compared in Figure 4b,c. Full model outputs for the linear *f*_org_ parameterization are shown in Figures S1 and S2.

### 3.1 Sensitivity tests

**FIGURE 5:**
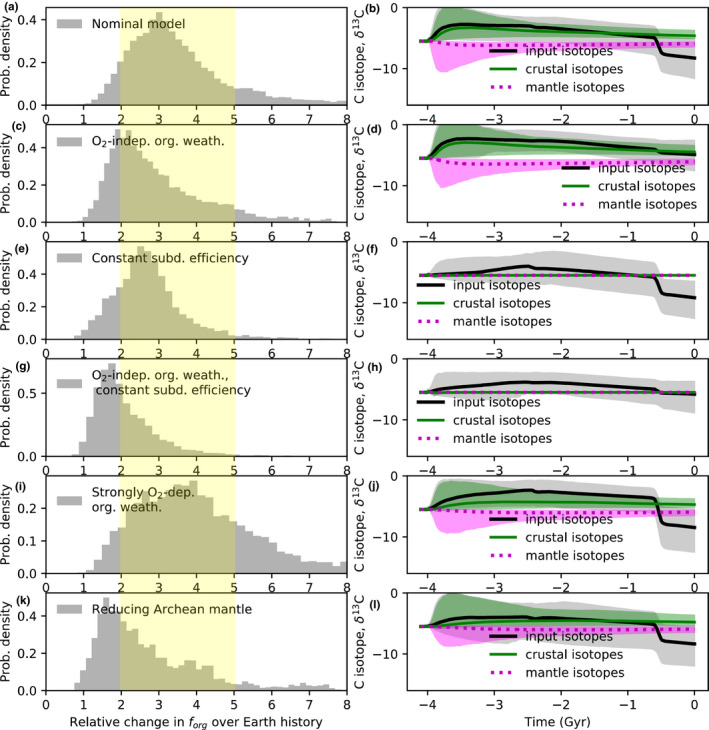
Sensitivity tests showing why our results differ from conventional interpretations of the carbon isotope record. Subplots on the left‐hand side denote the change in fractional organic burial over Earth history for various sensitivity tests, and the yellow shaded region shows the likely 2–5 fold change (1σ) from our nominal model (a). Subplots on the right‐hand side show the corresponding time evolution of the $\delta^{13}C$ of the mantle (magenta), crustal (red), and inputs (black–gray) to the atmosphere–ocean system (outgassing + carbonate weathering + organic weathering) for each sensitivity test. Lines denote median model outputs and shaded regions denote 95% credible intervals. Subplots (a) and (b) represent the nominal model, (c) and (d) assume organic whether is independent of atmospheric oxygen, (e) and (f) assume constant subduction efficiencies over Earth history, and (g) and (h) assume both oxygen‐independent organic weathering and constant subduction efficiencies. The results from the (h) sensitivity test are the most similar to conventional interpretations of the carbon isotope record because they ensure constant carbon isotopic inputs over Earth history. Subplots (i) and (j) show the opposite effect: strongly oxygen‐dependent organic weathering results in large inferred changes in fractional organic burial over Earth history due to large changes in carbon isotopic inputs. Finally, subplots (k) and (l) show results from assuming a more reduced Archean mantle (see Section 3.4). Note that for (e), (f), (g), and (h), mantle and crustal reservoirs through time remain approximately constant because carbon outputs via carbonate and organic burial are balanced by proportional return of carbonate and organics via arc volcanism and metamorphism. However, introducing preferential subduction of organic carbon unbalances outputs and inputs and allows crustal reservoirs to become isotopically heavier at the expense of the mantle

We repeated our inverse analyses for several endmember cases to elucidate which constraints are driving our results. Figure 5, left column, shows the inferred probability distributions for the change in fractional organic burial over Earth history for six different cases. Figure 5, right column, shows the isotopic evolution of the mantle, crust, and the carbon inputs in the atmosphere–ocean system (weathering + outgassing) for each of these scenarios.

Figure 5a shows the nominal model, where a 2–5 fold increase (1σ) in fractional organic burial over Earth history is inferred (highlighted). This is much larger than the inferred change in fractional organic burial inferred in previous work (e.g., Krissansen‐Totton et al., 2015), for two main reasons. First, the oxygen dependence of organic weathering causes a substantial shift in $\delta^{13}C_{\text{inputs}}$ in the Neoproterozoic; the isotopic composition of outgassing and weathering inputs shifts toward lighter values at this time due to an increase in organic weathering, which delivers isotopically light carbon to the atmosphere–ocean reservoir. If the inverse analysis is repeated but with organic weathering entirely independent of atmospheric oxygen (i.e., organic weathering is limited to the thermogenic component), then the inferred change in *f*_org_ over Earth history is smaller (Figure 5c,d). Second, the preferential subduction of organics in the Precambrian causes elevated crustal $\delta^{13}C$ values, and therefore heavier $\delta^{13}C_{\text{inputs}}$ in the Precambrian, which requires lower fractional organic burial to fit the isotope record.

We explicitly looked at the effect of subducted carbon in Figure 5e,f, where our inverse analysis is repeated with constant subduction efficiencies throughout Earth history. The combination of these two effects mostly explains why our results differ from previous analyses; Figure 5g,h show our inverse analysis repeated with O_2_‐independent organic weathering *and* constant subduction efficiency. Here, $\delta^{13}C_{\text{inputs}}$ remains approximately equal to mantle values throughout Earth history and the inferred *f*_org_ change is more closely aligned with previous studies. The median change in *f*_org_ in Figure 5g is 1.89, within error of the factor of $1.5_{‐0.3}^{+0.5}$ inferred in Krissansen‐Totton et al. (2015). Slight differences remain due to changing crustal reservoir sizes. In contrast, Figure 5i and 5j show results from a sensitivity test with strongly O_2_‐dependent organic weathering, that is, no thermogenic methane component, and all organic weathering is O_2_‐dependent. In this case, a larger change in fractional organic burial over Earth history is permitted and the change in $\delta^{13}C_{\text{inputs}}$ at the Neoproterozoic is larger than in the nominal model.

### 3.2 Reducing Archean mantle

The fit to data in Figures 2, 3, and 4 is marginally consistent with an anoxic Archean atmosphere; only 50% of nominal model runs in Figure 2h satisfy ${\text{K}}_{\text{oxy}}(\text{Archean})<1$. This suggests that while it is possible to construct an organic burial history consistent with the isotope record than can account for the anoxic–oxic transition at the GOE, declining oxygen sinks may have also contributed. Indeed, there is evidence for a secular oxidation of the upper mantle since the Archean, which would imply elevated fluxes of oxygen‐consuming gases on the early Earth (Aulbach & Stagno, 2016; Kadoya et al., 2020; Nicklas et al., 2019).

**FIGURE 6:**
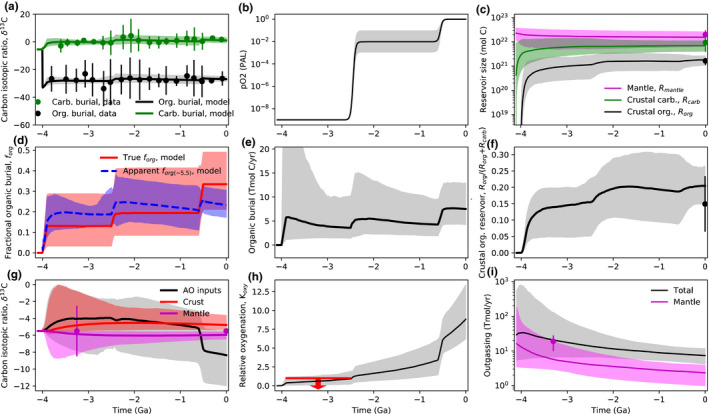
Inverse carbon cycle model outputs fit to data to constrain organic burial over Earth history with a reduced Archean mantle. In all subplots, solid lines denote median model outputs and shaded regions denote 95% credible intervals, whereas dots with error bars are empirical constraints (Table 1). Subplots are the same as in Figure 2. A reduced mantle helps ensure an anoxic Archean atmosphere (h) where K_oxy_ < 1. Additionally, total Precambrian outgassing fluxes of carbon (i) are lower than in the nominal model because less total outgassing is required to produce an anoxic Archean. Lower total carbon throughput means the absolute organic burial flux (e) is lower than the nominal model, despite the possibility of higher Precambrian fractional organic burial (d). This reduced Archean mantle scenario is consistent with the carbon isotope record (a) and with modern constraints on carbon reservoirs (c, f)

In Figure 6, we show the results of our inverse analyses where we impose a secular increase in mantle oxygen fugacity based on measured V/Sc ratios in mid ocean ridge basalts (Aulbach & Stagno, 2016). A 1.2 log‐unit change in mantle oxygen fugacity from the Archean to present has the effect of increasing the Archean reduced gas flux by a factor of approximately 3.5, which we parameterized as a term controlling the reduced gas flux in Equation 9:

(13) $$F_{\text{reduced}}=2.4\times 10^{12}\left(1+\frac{2.5t_{\mathrm{Gyr}}}{4.1}\right)$$

Introducing a reduced Archean mantle has several effects on the inverse analysis. First, the Archean anoxic constraint is satisfied much more comfortably (Figure 6h). Whereas only 50% of nominal model runs (Figure 2h) satisfy ${\text{K}}_{\text{oxy}}(\text{Archean})$ < 1.0, with a reducing mantle this increases to 80% (Figure 6h). A greater flux of reduced gases implies the absolute carbon outgassing flux may be lower than in the nominal case since less total carbon outgassing is required for the same amount of reducing power (Figure 6i). This, in turn, lowers absolute organic burial (Figure 6e). Crucially, there need not be such a large change in fractional organic burial to explain the transition from anoxic‐to‐oxic atmosphere (Figure 5k), although large changes in fractional organic burial are still permitted.

### 3.3 Land fraction and weathering function dependence

**FIGURE 7:**
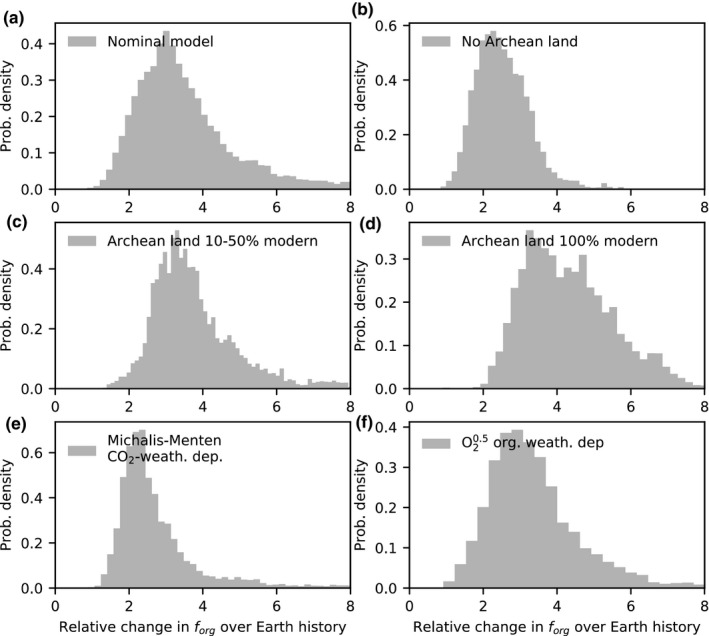
Sensitivity tests showing how our results depend on continental growth and functional forms for carbonate and organic weathering. Distribution for the change in fractional organic burial over Earth history is plotted for (a) nominal model, (b) no Archean land, (c) Archean land fraction 10%–50% of modern, (d) Archean land fraction constant over Earth history, (e) a Michaelis–Menten law for the atmospheric CO_2_ dependence of continental weathering, and (f) a stronger oxygen dependence for organic weathering. The key result here is that lower Archean land results in lower inferred changes in fractional organic burial over Earth history, whereas weathering parameterizations have a relatively minor effect on organic burial changes

Figure 7 shows the sensitivity or our results to different assumed land fraction evolutions and functional forms for carbonate and organic weathering. Although there is evidence for the early emplacement of continents (Rosas & Korenaga, 2018), it is challenging to reconstruct land fraction from continental growth curves due to uncertainties in the deep hydrological cycle (Korenaga, 2018). Figure 7a shows the inferred fractional organic burial change from our nominal model (0%–50% Archean land fraction), whereas Figure 7b shows results from a repeated analysis with negligible Archean land. Figure 7c,d show repeated analyses for Archean land that is 10%–50% modern and 100% modern, respectively. The broad trend is that lower Archean land fractions imply smaller changes in fractional organic burial over Earth history. This is because carbonate weathering is assumed to scale with the land fraction, and so lower land fractions yield smaller Precambrian carbonate weathering fluxes. This has the effect of partially offsetting the preferential subduction of organic matter in the Archean and producing more constant $\delta^{13}C_{\text{inputs}}$ over Earth history. Carbonate weathering fluxes also grow more dramatically with continental growth, compensating for the increase in organic weathering triggered by increasing atmospheric oxygen.

A similar effect can be seen in Figure 7e where our analysis was repeated assuming a Michaelis–Menten CO_2_ dependence for carbonate weathering:

(14) $$F_{\text{Weath\_carb}}=F_{\text{Weath\_carb}}^{\text{modern}}\left(\frac{R_{\text{carb}}}{R_{\text{carb}}^{\text{modern}}}\right)\left(\frac{f_{\text{land}}}{f_{\text{land}}^{\text{modern}}}\right){\left(\frac{2{\text{pCO}}_{2}/{\text{pCO}}_{2}^{\text{modern}}}{1+{\text{pCO}}_{2}/{\text{pCO}}_{2}^{\text{modern}}}\right)}^{\alpha }\exp\left(\frac{\Delta T_{S}}{T_{e}}\right)$$

here, α is an unknown exponent with a prior range from 0 to 1. In this scenario, carbonate weathering is more weakly dependent on atmospheric CO_2_, and so carbonate weathering fluxes are lower in the Archean when atmospheric CO_2_ was necessarily high due the silicate‐weathering thermostat (Krissansen‐Totton et al., 2018). This has the effect of ensuring smaller changes in $\delta^{13}C_{\text{inputs}}$ over Earth history, and so higher fractional organic burial is required in the Precambrian to fit the isotope record. Figure 7f shows the impact of a stronger pO_2_ dependence for oxidative weathering (Daines et al., 2017). In this case, the impacts on the inferred fractional organic burial change over Earth history are minor. Broadly speaking, these tests show that while continental growth and the functional dependence of carbonate and organic weathering have some impact on the inferred changes in organic burial over Earth history, fractional organic burial likely must have increased by a factor of a few to fit the carbon isotope record.

## 4 DISCUSSION

A previous analysis of the carbon isotope record implied that *f*_org_ increased by approximately ~1.5 over Earth history (Krissansen‐Totton et al., 2015), but that study assumed that $\delta^{13}C$ of carbon inputs equaled constant (modern) mantle values whereas the present study does not. The inverse analysis in this study reveals that somewhat larger changes in fractional organic burial are compatible with the carbon isotope record, potentially a $3.2_{‐0.9}^{+1.5}$ (1σ) fold increase over Earth history (Figure 4a). This change in organic burial is consistent with a transition from an anoxic‐to‐oxic atmosphere in Paleoproterozoic (Figures 2h and 3f). To be clear, our results are agnostic as to why fractional organic burial has increased over Earth history; the increase in *f*_org_ we infer is simply the trend required to fit the carbon isotope record. Moreover, our results do not imply that fractional organic burial increases were the only cause of Earth's atmospheric oxygenation. Indeed, it is likely that declining oxygen sinks also contributed to the rise of oxygen, as illustrated in Figure 6. What our calculations do demonstrate is that the relatively invariant carbon isotope record does not necessarily imply a fixed organic burial fraction over Earth history, and that changes in fractional organic burial large enough to account for the anoxic–oxic transition can be reconciled with both the isotope record and other geologic constraints.

This overall 2‐ to 5‐fold increase in fractional organic carbon burial can be decomposed into an increase between the Proterozoic and Archean (Figure 4b), and a larger increase between the Proterozoic and the Phanerozoic (Figure 4c). The latter increase is when changes in atmospheric oxygen have the largest effect on oxidative weathering fluxes, and therefore on $\delta^{13}C_{\text{inputs}}$. This is because even though the relative change in atmospheric oxygen is greater across the Archean–Proterozoic transition than the Proterozoic–Phanerozoic transition, changes in absolute flux govern isotopic mass balance and redox evolution. The exponential oxygen dependence in Equation (6) results in a larger absolute change in organic weathering across the Proterozoic–Phanerozoic transition. In absolute terms, the oxidative weathering flux is small in the Mesoproterozoic, and so the transition from negligible oxidative weathering in the Archean to small oxidative weathering in the Mesoproterozoic does not dramatically modify carbon weathering inputs compared to the Neoproterozoic transition. This suggests that enhanced organic burial likely played an important role in the stepwise increases in oxygen during Neoproterozoic and/or Paleozoic oxygenation, although we do not explicitly model oxygen cycling in this study and do not attempt to disentangle cause and effect in the Neoproterozoic.

If the pO_2_ dependence of organic weathering and thermogenic CH_4_ fluxes was known more precisely, then we could better constrain Earth's fractional organic burial record (see Figure 5). Exactly how much thermogenic CH_4_ was produced in the Archean is uncertain. Oil and petroleum production from thermal maturation of organic‐rich shales clearly occurred (Mossman et al., 2008; Rasmussen, 2005), and black shale deposition may have been extensive in the late Archean (Condie et al., 2001).

Somewhat lower *f*_org_ in the Archean helps to explain the paradox of high rates of total carbon burial in the Archean despite evidence for lower primary productivity (see Introduction). However, it is unlikely that the changes in fractional organic burial implied by this study could resolve the paradox entirely. This is because even though Archean fractional organic burial may have been lower, the absolute Archean organic burial flux in our inverse model is comparable to the modern flux (Figure 2e) as total carbon throughput is high due to elevated Precambrian outgassing (Figure 2i). This high throughput is supported by a 3.3 Ga constraint on mantle outgassing from radiogenic ^129^Xe (Avice et al., 2017). In our model, early C outgassing is parameterized to be high via scaling with relative heat flow. Changes in organic burial efficiency are likely also required to explain lower Precambrian productivity, as explored in the next section.

Our nominal model suggests a modest increase in the crustal organic reservoir from the Archean to the present (Figure 2c), in large part due to the relative constancy of the absolute organic burial flux (Figure 2e). This is broadly consistent with the measured organic carbon content of Archean marine sedimentary rocks, which is indistinguishable from that of recent sediments (Krissansen‐Totton et al., 2015; Lyons et al., 2014). While assessing total marine sediment inventories is challenging, our model results are also broadly in agreement with more detailed reconstructions of sedimentary total organic carbon (TOC) content across geologic time. For instance, Sperling and Stockey (2018, their Figure 2) show distributions of TOC content for Proterozoic and Phanerozoic sediments. Although our model is not designed to capture variations in organic reservoirs within the Proterozoic with our assumed *f*_org_ parameterization, the difference between the TOC content in the Phanerozoic and mean Proterozoic from Sperling and Stockey (2018, their Figure 2) is consistent with the organic reservoir evolution in our model.

Note that in our nominal model, fractional organic burial and the organic‐to‐total‐carbon fraction in the crust need not be equal because isotopic inputs into the atmosphere–ocean can diverge from mantle values, and because organics are preferentially subducted into the mantle. Our results are therefore able to reconcile a relatively high modern *f*_org_ = 0.32 ± 0.07 (Figure 2d) with a crustal organic fraction of roughly 0.15 ± 0.08 (Figure 2f). This may help resolve the discrepancy between crustal organic contents and organic burial rates reviewed in Derry (2014).

### 4.1 Implications for Precambrian burial efficiency

**FIGURE 8:**
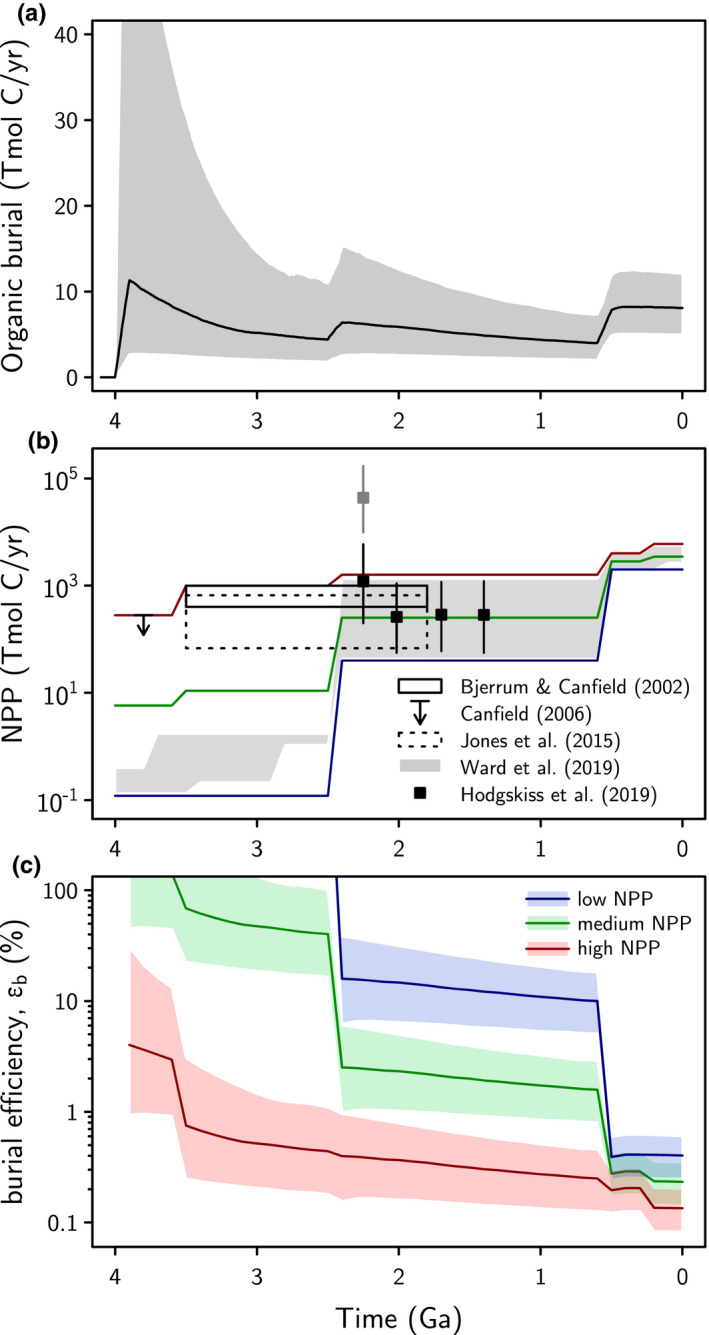
Implications for organic burial efficiency. (a) shows absolute organic carbon burial flux from our nominal model, (b) shows various net primary productivity (NPP) constraints from the literature (see main text). Three plausible scenarios for high (red), medium (green), and low (blue) NPP are plotted alongside proposed constraints for reference. These scenarios and the 95% envelope in (a) are combined to calculate the required organic burial efficiency for each scenario in subplot (c). High–Medium Archean primary productivity requires burial efficiencies between a few percent to a few tens of percent

Invariant absolute organic burial requires that Archean organic burial efficiency must have been much higher than average modern burial efficiency (e.g., Kipp & Stüeken, 2017; Laakso & Schrag, 2018) to allow for lower primary productivity. In Figure 8, we compare our inferred absolute organic burial flux from our nominal model (Figure 8a) to various literature estimates of net primary productivity (NPP) through time (Figure 8b). These include constraints derived from estimated reductant fluxes in the pre‐oxygenic photosynthesis biosphere (Canfield et al., 2006; Ward et al., 2019), nutrient availability (namely phosphorus) in the post‐oxygenic photosynthesis world (Bjerrum & Canfield, 2002; Jones et al., 2015), and rare oxygen isotope anomalies that track biospheric oxygen production (Crockford et al., 2018; Hodgskiss et al., 2019); a thorough discussion of these constraints can be found in Kipp et al. (2021). We summarize these productivity reconstructions with “High,” “Medium,” and “Low” NPP scenarios, which are subsequently combined with our absolute organic burial envelopes to infer burial efficiency (Figure 8c):

(15) $$\text{Burial}\;\text{efficiency},\;\varepsilon_{b}\;=\;\frac{F_{\text{Burial}\_\text{org}}}{\text{NPP}}$$

Figure 8c shows that our inferred organic burial flux, when divided by a modern NPP estimate of 4,000 Tmol C/year (Field et al., 1998), implies a modern burial efficiency of around 0.1%–0.5%, consistent with traditional literature estimates (e.g., Hedges & Keil, 1995; Holland, 1984). Moreover, it shows that Archean primary productivity 1–2 orders of magnitude lower than today would require burial efficiencies of a few percent to tens of percent, as suggested by simpler calculations in Kipp et al. (2021). For comparison, organic burial efficiency in anoxic Black Sea sediments is around 2% (Arthur et al., 1994), whereas in anoxic and low‐sulfate Lake Matano burial efficiency may be several tens of percent (Kuntz et al., 2015). The calculations in Figure 8 were repeated for the reduced Archean mantle case in Figure S5 and the conclusions are unchanged.

Broadly speaking, our inferred organic burial fluxes would seem to exclude very low Archean NPP estimates (*i.e*., several orders of magnitude lower than modern), such as those from Ward et al. (2019), because they require burial efficiency greater than 100%. Coupled ecosystem–climate models of chemoautotrophic ecosystems imply primary productivity 4–7 orders of magnitude lower than that of the modern Earth (Sauterey et al., 2020). Such low primary productivity is also incompatible with the Archean carbon isotope record (Figure 8c), suggesting that nutrient‐limited photosynthetic organisms likely dominated global primary production since at least 3.5 Ga (Sauterey et al., 2020). This is consistent with geologic evidence for stromatolites at 3.5 Ga (Walter et al., 1980).

### 4.2 Choice of absolute fluxes and the possibility of high modern *f*_org_

Here, we explore the sensitivity of our results to assumed priors for modern organic and carbonate weathering fluxes (see Section 2.6 for a discussion of different absolute flux estimates). Figure S4 shows our nominal model ($F_{\text{oxid}}^{\text{modern}}+F_{\text{thermo}}^{\text{modern}}$ = 3–9 Tmol C/year, $F_{\text{Weath\_carb}}^{\text{modern}}$ = 7–25 Tmol C/year), a sensitivity test with high modern organic weathering and low modern carbonate weathering ($F_{\text{oxid}}^{\text{modern}}+F_{\text{thermo}}^{\text{modern}}$ = 6–12 Tmol C/year, $F_{\text{Weath\_carb}}^{\text{modern}}$ = 7–15 Tmol C/year), and a sensitivity test with low modern organic weathering and high modern carbonate weathering ($F_{\text{oxid}}^{\text{modern}}+F_{\text{thermo}}^{\text{modern}}$ = 3–9 Tmol C/year, $F_{\text{Weath\_carb}}^{\text{modern}}$ = 15–25 Tmol C/year). These calculations show that the choice of prior does not significantly impact the inferred *relative change* in fractional organic burial over Earth history. The result that fractional organic burial has increased by 2–5 times over Earth history is robust to choices of absolute fluxes.

However, the choice of modern fluxes does impact the *absolute* value for modern fractional organic burial. In our nominal model, modern *f*_org_ is 0.32 (95% credible range 0.20–0.45), which is broadly consistent with the canonical value of 0.2–0.3 (Derry, 2014; Hayes et al., 1999; Rothman, 2015). Our median model value is slightly larger than the canonical range because we allow $\delta^{13}C_{\text{inputs}}$ to diverge from mantle values as organic weathering increases. For the case of high modern organic weathering and low modern carbonate weathering, modern *f*_org_ is 0.45 (95% credible 0.32–0.55). Such a high value for fractional organic burial would have implications for the modern redox budget, and so it is worth considering the plausibility of this result.

We disfavor high modern *f*_org_ because the incomplete oxidation of organic matter in the modern environment tentatively suggests high modern organic weathering fluxes are unlikely (see Methods). However, numerous studies argue for high modern organic burial fluxes (e.g., Holland, 2002), and the high modern fractional organic burial scenario satisfies all constraints in Table 1 (see Figure S6). Specifically, modern *f*_org_ = 0.45 is compatible with modern crustal reservoirs, including a ~0.15 organic carbon to total carbon crustal inventory ratio (Figure S6), due to the preferential subduction of organics. There is no conflict between *f*_org_ = 0.45 and canonical interpretations of the carbon isotope record that assume inputs have always been isotopically identical to the mantle, that is, apparent *f*_org_ (−5.5) = 0.24 in the Phanerozoic despite *f*_org_ = 0.45 (Figure S6).

One possible inconsistency, however, arises from independent constraints on $\delta^{13}C_{\text{inputs}}$. Model values for modern $\delta^{13}C_{\text{inputs}}$ (−9‰ to −14‰) in the high *f*_org_ scenario differ from Cenozoic $\delta^{13}C_{\text{inputs}}$ inferred from regression analyses of pairs of $\delta^{13}C_{\text{Burial\_carb}}$ and $\delta^{13}C_{\text{Burial\_carb}}‐\delta^{13}C_{\text{Burial\_org}}$ data, −2.8‰ to −7.6‰ (Derry, 2010; Rothman et al., 2003). At other times, such as the Cambrian–Precambrian boundary, carbon inputs range from −7.1‰ to −13.2‰ (Derry, 2010, 2014). While modern $\delta^{13}C_{\text{inputs}}$ in our nominal model (−5.1‰ to −11.6‰ with 95% confidence) is comfortably within error of both of these estimates, the high modern *f*_org_ scenario potentially conflicts with the Cenozoic data. On the other hand, the data set used to infer the latter is small, and inferred $\delta^{13}C_{\text{inputs}}$ values are highly sensitive to small changes in the chosen data set, so the possibility of high fractional organic burial in the modern cannot be excluded. One opportunity for future work would be a more comprehensive regression analysis of Phanerozoic carbon isotope data to constrain $\delta^{13}C_{\text{inputs}}$. Additionally, better constraints on the incomplete oxidation of organic matter and global organic weathering and carbonate fluxes would help constrain modern *f*_org_ values.

The absolute modern organic burial flux in our nominal model is 5–13 Tmol C/year (95% credible interval), which is in agreement with literature estimates that typically range from 5 to 10 Tmol/year (Catling & Kasting, 2017; Holland, 2002; Lenton et al., 2018). In summary, the sensitivity tests presented here show that the decoupling between the carbon isotope record and organic burial in our model potentially allows for high modern fractional organic burial. However, improved constraints on absolute fluxes would be necessary to assess this possibility.

### 4.3 Additional caveats

Finally, we did not consider missing carbon sinks in our analysis such as authigenic carbonates (Schrag et al., 2013) or seafloor carbonates with different $\delta^{13}C$ values to shelf carbonates (Bjerrum & Canfield, 2004). However, Archean carbonates show no obvious depth gradient (Krissansen‐Totton et al., 2015; Nakamura & Kato, 2004). The inclusion of authigenic carbonates could potentially allow for even larger changes in fractional organic burial over Earth history, but the size of this hypothetical missing sink over time has no empirical quantitative constraints for us to consider. Although there are preservation biases to consider, carbonate concretions are seemingly rare in the Archean—suggesting little role for authigenic carbonates—and they increase after 2 Ga and further in the Phanerozoic (Fallick et al., 2008). Consequently, if authigenic carbonates are a relatively minor flux today (Sun & Turchyn, 2014), then there is no empirical reason to think there were important in the past when signs for such carbonates are rarer (Fallick et al., 2008). Our model also ignored any oxygen dependence of the $\delta^{13}C$ of new organic matter (c.f. Tappert et al., 2013) and treated crustal and mantle reservoirs as well‐mixed and isotopically homogeneous.

## 5 CONCLUSIONS

Self‐consistent carbon cycle modeling of the mantle, crust, and surface reservoirs shows that the $\delta^{13}C$ of carbon inputs into the atmosphere and oceans need not equal mantle values over Earth history. This implies that fractional organic burial may not be straightforwardly reflected in the carbon isotope record. In fact, inverse analyses using this carbon cycle model reveal that a 2‐ to 5‐fold increase (1σ) in fractional organic burial over Earth history is consistent with the carbon isotope record, modern carbon cycle constraints, and an anoxic Archean atmosphere. This change in fractional organic burial can potentially account for the transition from anoxic‐to‐oxic atmosphere without recourse to declining oxygen sinks, although declining oxygen sinks due to a more reduced Archean mantle help guarantee an anoxic–oxic transition. The absolute Archean organic burial flux was potentially comparable to the modern burial flux, although the uncertainty in total carbon throughput is large and strongly model dependent. If absolute Archean organic burial was large, then a necessarily elevated Archean burial efficiency would explain low Precambrian productivity and rule out extremely low Archean primary productivity.

## CONFLICT OF INTEREST

The authors declare no conflict of interest.

## Supporting information

Supplementary Material

## Data Availability

The Python code used for this analysis is available on the lead author's Github upon publication: https://github.com/joshuakt/Carbon‐isotopes‐inverse‐model
