# Supplementary material for: Carbon cycle inverse modeling suggests large changes in fractional organic burial are consistent with the carbon isotope record and may have contributed to the rise of oxygen
Source: Geobiology. 2021 Mar 25;19(4):342–63. doi: 10.1111/gbi.12440 (PMC8359855; doi:10.1111/gbi.12440)
Supplement: Supplementary file 1 — Supplementary Material [file GBI-19-342-s001.pdf]

Supporting Information for

Carbon cycle inverse modeling suggests large changes in fractional organic burial are consistent with the carbon isotope record and may have contributed to the rise of oxygen

Contents of this file

Text S1 to S5

Figures S1 to S9

Introduction

Supplementary Text S1 to S5 describes the details of our carbon cycle model. The python code is available upon publication. Supplementary Fig. S1-S6 are sensitivity tests as described in the main text, whereas Fig. S7-S9 illustrate mass conservation and other model properties.

Text S1: Detailed model description

We relate the carbon outgassing component from the mantle, $F_{\text{out\_mantle}}$ (mol C/yr), to the relative internal heatflow, Q (dimensionless), and mantle reservoir size, R_{mantle} (mol C), as follows:

$$F_{\text{out\_mantle}} = F_{\text{out\_mantle}}^{modern} \times Q^{\mu} \times R_{mantle} / R_{mantle}^{modern} \tag{1}$$

Here $F_{\text{out-mantle}}^{\text{mod}}$ is the modern carbon outgassing flux from the mantle (see below), and μ is a constant between 0.0 and 2.0. Relative heatflow (dimensionless) is given by $Q = (1 - t_{Gyr}/4.5)^{n_{out}}$, where n_{out} is some exponent between 0 and 2.0, and t_{Gyr} (Ga) is the time before the present.

The subduction and metamorphic fluxes of carbon (mol C/yr) are defined as follows:

$$\begin{aligned}
F_{\text{sub_carb}} &= F_{\text{sub_carb}}^{modern} \times r_{\text{spread-rate}} \times R_{carb} / R_{carb}^{modern} \\
F_{\text{sub_org}} &= F_{\text{sub_org}}^{modern} \times r_{\text{spread-rate}} \times R_{org} / R_{org}^{modern} \\
F_{\text{meta_carb}} &= F_{\text{meta_carb}}^{modern} \times Q^{\mu} \times R_{carb} / R_{carb}^{modern} \\
F_{\text{meta_org}} &= F_{\text{meta_org}}^{modern} \times Q^{\mu} \times R_{org} / R_{org}^{modern}
\end{aligned} \tag{2}$$

Here, the relative spreading rate (dimensionless) is $r_{\text{spread-rate}} = Q^{\beta}$ where β is an exponent between 1 and 2. Modern fluxes for the subduction of carbonates ($F_{\text{sub\_carb}}^{modern}$), the subduction of organics ($F_{\text{sub\_org}}^{modern}$), the metamorphism of carbonates ($F_{\text{meta\_carb}}^{modern}$), and the metamorphism of organics ($F_{\text{meta\_org}}^{modern}$) are defined below. Continental and seafloor weathering fluxes of silicates have the following parameterizations:

$$F_{\text{sil}} = F_{\text{sil}}^{modern} \left(\frac{f_{\text{land}}}{f_{\text{land}}^{modern}} \right) \left(\frac{f_{\text{bio}}}{f_{\text{bio}}^{modern}} \right) \left(\frac{\text{pCO}_2}{\text{pCO}_2^{modern}} \right)^{\alpha} \exp\left(\frac{\Delta T_S}{T_e} \right) \tag{3}$$

$$F_{\text{dis}} = F_{\text{dis}}^{modern} r_{\text{spread-rate}} \exp\left[-\frac{E_{\text{bas}}}{R} \left(\frac{1}{T_{pore}} - \frac{1}{T_{pore}^{modern}} \right) \right] \left(\frac{[H^+]}{[H^+]^{modern}} \right)^{\gamma} \tag{4}$$

Here, F_{sil} (mol C/yr) is the silicate weathering flux on land, and F_{dis} is the dissolution of seafloor basalt (mol C/yr). The temperature dependence of continental silicate weathering is governed by the temperature difference with modern mean surface temperature divided by an e-folding temperature, $\Delta T_S / T_e$, and the CO_2-dependence is governed by an exponent, α . The temperature dependence of

seafloor weathering is governed by the effective activation energy, E_{bas} (kJ/mol), and the ocean pH dependence (or more specifically H+(aq) concentration dependence) is governed by the exponent γ = 0.25. Assumed parameter ranges for these unknown variables are shown in Table 2, and R (0.008314 kJ/mol/K) is the universal gas constant. Seafloor dissolution also depends on the relative spreading rate, $r_{\text{spread-rate}}$, defined above. Continental silicate weathering depends on the subaerial land fraction relative to modern, $f_{\text{land}} / f_{\text{land}}^{modern}$, and on the biological enhancement of weathering, $f_{\text{bio}} / f_{\text{bio}}^{modern}$, both of which are described in Krissansen-Totton et al. (2018).

Carbonate precipitation fluxes in the ocean and the pore space, P_{ocean} and P_{pore}, respectively, depend on the saturation state of the ocean, as described in Krissansen-Totton and Catling (2017). Note that the total carbonate burial flux is the sum of these two terms. Initialization and modern flux values are described below.

Text S2: Carbon isotope evolution

The model tracks the time-evolution of carbon isotopes using the following system of equations. The evolution of $\delta^{13}\text{C}$ of crustal organic carbon, $\delta^{13}\text{C}_{\text{org}}$, is given by:

$$\frac{d(\delta^{13}\text{C}_{\text{org}} R_{org})}{dt} = F_{\text{Burial\_org}}\delta^{13}\text{C}_{\text{org\_buried}} - F_{\text{sub\_org}}\delta^{13}\text{C}_{\text{org}} - F_{\text{meta\_org}}\delta^{13}\text{C}_{\text{org}} - F_{\text{Weath\_org}}\delta^{13}\text{C}_{\text{org}}$$

$$\frac{d\delta^{13}\text{C}_{\text{org}}}{dt} = -\frac{\delta^{13}\text{C}_{\text{org}}}{R_{org}}\frac{dR_{org}}{dt} + \frac{(F_{\text{Burial\_org}}\delta^{13}\text{C}_{\text{org\_buried}} - F_{\text{sub\_org}}\delta^{13}\text{C}_{\text{org}} - F_{\text{meta\_org}}\delta^{13}\text{C}_{\text{org}} - F_{\text{Weath\_org}}\delta^{13}\text{C}_{\text{org}})}{R_{org}} \tag{5}$$

The evolution of $\delta^{13}C$ of crustal carbonate carbon:

$$\frac{d(\delta^{13}\text{C}_{\text{carb}} R_{carb})}{dt} = P_{\text{ocean}}\delta^{13}\text{C}_{\text{precip}} + P_{\text{pore}}\delta^{13}\text{C}_{\text{precip}} - F_{\text{Weath\_carb}}\delta^{13}\text{C}_{\text{carb}} - F_{\text{sub\_carb}}\delta^{13}\text{C}_{\text{carb}} - F_{\text{meta\_carb}}\delta^{13}\text{C}_{\text{carb}}$$

$$\frac{d\delta^{13}\text{C}_{\text{carb}}}{dt} = -\frac{\delta^{13}\text{C}_{\text{carb}}}{R_{carb}}\frac{dR_{carb}}{dt} + \frac{(P_{ocean}\delta^{13}\text{C}_{\text{precip}} + P_{\text{pore}}\delta^{13}\text{C}_{\text{precip}} - F_{\text{Weath\_carb}}\delta^{13}\text{C}_{\text{carb}} - F_{\text{sub\_carb}}\delta^{13}\text{C}_{\text{carb}} - F_{\text{meta\_carb}}\delta^{13}\text{C}_{\text{carb}})}{R_{carb}}$$

(6)

The evolution of the $\delta^{13}C$ of mantle carbon:

$$\frac{d(\delta^{13}\mathrm{C}_{\mathrm{mantle}}R_{mantle})}{dt}=\xi_{carb}F_{\mathrm{sub\_carb}}\delta^{13}\mathrm{C}_{\mathrm{carb}}+\xi_{org}F_{\mathrm{sub\_org}}\delta^{13}\mathrm{C}_{\mathrm{org}}-F_{\mathrm{out\_mantle}}\delta^{13}\mathrm{C}_{\mathrm{mantle}}$$

$$\frac{d\delta^{13}\mathrm{C}_{\mathrm{mantle}}}{dt}=-\frac{\delta^{13}\mathrm{C}_{\mathrm{mantle}}}{R_{mantle}}\frac{dR_{mantle}}{dt}+\frac{\xi_{carb}F_{\mathrm{sub\_carb}}\delta^{13}\mathrm{C}_{\mathrm{carb}}+\xi_{org}F_{\mathrm{sub\_org}}\delta^{13}\mathrm{C}_{\mathrm{org}}-F_{\mathrm{out\_mantle}}\delta^{13}\mathrm{C}_{\mathrm{mantle}}}{R_{mantle}} \quad (7)$$

$$\frac{d\delta^{13}\mathrm{C}_{\mathrm{mantle}}}{dt}=-\frac{\delta^{13}\mathrm{C}_{\mathrm{mantle}}}{R_{mantle}}\frac{dR_{mantle}}{dt}+\frac{(\xi_{carb}F_{\mathrm{sub\_carb}}\delta^{13}\mathrm{C}_{\mathrm{carb}}+\xi_{org}F_{\mathrm{sub\_org}}\delta^{13}\mathrm{C}_{\mathrm{org}}-F_{\mathrm{out\_mantle}}\delta^{13}\mathrm{C}_{\mathrm{mantle}})}{R_{mantle}}$$

The evolution of the $\delta^{13}C$ of the carbon in the atmosphere-ocean reservoir:

$$\frac{d(\delta^{13}\mathrm{C}_{\mathrm{AO}}R_{AO})}{dt}=F_{\mathrm{outg}}\delta^{13}\mathrm{C}_{\mathrm{outg}}+F_{\mathrm{Weath\text{-}carb}}\delta^{13}\mathrm{C}_{\mathrm{carb}}+F_{\mathrm{Weath\text{-}org}}\delta^{13}\mathrm{C}_{\mathrm{org}}$$
$$-P_{\mathrm{ocean}}\delta^{13}\mathrm{C}_{\mathrm{precip}}-P_{precip}\delta^{13}\mathrm{C}_{\mathrm{precip}}-F_{\mathrm{burial\text{-}org}}\delta^{13}\mathrm{C}_{\mathrm{org\text{-}buried}}$$
$$\frac{d\delta^{13}\mathrm{C}_{\mathrm{AO}}}{dt}=-\frac{\delta^{13}\mathrm{C}_{\mathrm{AO}}}{R_{AO}}\frac{dR_{AO}}{dt}$$
$$+\frac{F_{\mathrm{outg}}\delta^{13}\mathrm{C}_{\mathrm{outg}}+F_{\mathrm{Weath\text{-}carb}}\delta^{13}\mathrm{C}_{\mathrm{carb}}+F_{\mathrm{Weath\text{-}org}}\delta^{13}\mathrm{C}_{\mathrm{org}}-P_{\mathrm{ocean}}\delta^{13}\mathrm{C}_{\mathrm{precip}}-P_{\mathrm{precip}}\delta^{13}\mathrm{C}_{\mathrm{precip}}-F_{\mathrm{burial\text{-}org}}\delta^{13}\mathrm{C}_{\mathrm{org\text{-}buried}}}{R_{AO}}$$

(8)

Here, $\delta^{13}\mathrm{C}_{\mathrm{AO}}$ (‰) is the isotopic ratio of atmosphere-ocean carbon, and R_{AO} (mol C) is the size of the atmosphere-ocean reservoir. Additionally, we have the following definitions for the average carbon isotope value of outgassed carbon and crustal carbon, respectively:

$$\delta^{13}\mathrm{C}_{\mathrm{outg}}=\left(\frac{F_{\mathrm{out\_mantle}}\delta^{13}\mathrm{C}_{\mathrm{mantle}}+F_{\mathrm{meta\_carb}}\delta^{13}\mathrm{C}_{\mathrm{carb}}+F_{\mathrm{meta\_org}}\delta^{13}\mathrm{C}_{\mathrm{org}}+(1-\xi_{carb})F_{\mathrm{sub\_carb}}\delta^{13}\mathrm{C}_{\mathrm{carb}}+(1-\xi_{org})F_{\mathrm{sub\_org}}\delta^{13}\mathrm{C}_{\mathrm{org}}}{F_{\mathrm{out\_mantle}}+F_{\mathrm{meta\_carb}}+F_{\mathrm{meta\_org}}+(1-\xi_{carb})F_{\mathrm{sub\_carb}}+(1-\xi_{org})F_{\mathrm{sub\_org}}}\right)$$

$$\delta^{13}\mathrm{C}_{\mathrm{crust}}=\frac{R_{org}\delta^{13}\mathrm{C}_{\mathrm{org}}+R_{carb}\delta^{13}\mathrm{C}_{\mathrm{carb}}}{R_{org}+R_{carb}}$$

(9)

Carbon isotope fractionation is introduced as follows, where an average 28‰ fractionation between organic carbon and carbonate carbon burial is imposed (Krissansen-Totton et al. 2015):

$$\begin{aligned} \delta^{13}\mathrm{C}_{\mathrm{precip}} &= \delta^{13}\mathrm{C}_{\mathrm{AO}} + 2.0 \\ \delta^{13}\mathrm{C}_{\mathrm{org\_buried}} &= \delta^{13}\mathrm{C}_{\mathrm{precip}} - 28.0 \end{aligned} \tag{10}$$

Text S3: Model initialization and initial conditions

The general procedure for initializing the model at 4.1 Ga is outlined in this section. The code is available upon publication.

Step 1: Parameter ranges in Table 2 are sampled to obtain values for these 22 unknown variables.

Step 2: Modern metamorphic, subduction, and arc volcanism fluxes are assumed to be known:

$$\begin{aligned} &F_{\mathrm{out\_mantle}}^{modern} = 3\ \mathrm{Tmol\ C/yr},\ F_{\mathrm{meta}}^{modern} = 3\ \mathrm{Tmol\ C/yr} \\ &F_{\mathrm{sub}}^{modern} = 6\ \mathrm{Tmol\ C/yr} \\ &F_{\mathrm{meta\_carb}}^{modern} = F_{\mathrm{meta}}^{modern}\left(\frac{R_{carb}^{modern}}{R_{carb}^{modern} + R_{org}^{modern}}\right) \\ &F_{\mathrm{meta\_org}}^{modern} = F_{\mathrm{meta}}^{modern}\left(\frac{R_{org}^{modern}}{R_{carb}^{modern} + R_{org}^{modern}}\right) \\ &F_{\mathrm{sub\_carb}}^{modern} = F_{\mathrm{sub}}^{modern}\left(\frac{R_{carb}^{modern}}{R_{carb}^{modern} + R_{org}^{modern}}\right) \\ &F_{\mathrm{sub\_org}}^{modern} = F_{\mathrm{sub}}^{modern}\left(\frac{R_{org}^{modern}}{R_{carb}^{modern} + R_{org}^{modern}}\right) \\ &F_{\mathrm{arc\_mod}}^{modern} = (1-\xi_{carb}(modern))F_{\mathrm{sub\_carb}}^{modern} + (1-\xi_{org})F_{\mathrm{sub\_org}}^{modern} \end{aligned} \tag{11}$$

We do not propagate uncertainties in these values to avoid requiring an unwieldy number of unknown variables. However, it is the ratio of outgassing fluxes to weathering fluxes that is important for C isotope evolution, and by sampling a broad range of weathering fluxes, we are effectively accounting for the uncertainty in modern outgassing and subduction fluxes.

Step 3: Modern values for the total carbon inputs and total modern burial fluxes are calculated assuming the modern atmosphere-ocean reservoir is in steady state. Sampled prior values for the modern organic burial fraction, $j_1 j_2 j_3$, are also used in this calculation. The model run is discarded (i.e. the input parameters are assigned a negative infinity log-likelihood) if a negative continental silicate weathering flux is required to achieve steady state. Otherwise, the modern value for continental silicate weathering, $F_{\mathrm{sil}}^{modern}$, is determined by steady state requirements.

Step 4: Relative internal heatflow at 4.1 Ga, $Q\left(4.1\,Ga\right)$, is calculated from sampled value for n_{out}. If subduction efficiency is variable then an initial mantle temperature is also calculated. The heatflow at 4.1 Ga is then used to calculate initial metamorphic fluxes, subduction fluxes, and mantle outgassing using sampled values for μ, β, Hadean reservoirs, and equation (2).

Step 5: Given assumed initial pCO_2 and pH values at 4.1 Ga from Step 1, solve for the atmosphere-ocean chemical equilibrium, surface temperature (from the climate model described in Krissansen-Totton et al. (2018)) ,and continental land fraction as described in the main text and references therein.

Step 6: Calculate the continental silicate weathering flux at 4.1 Ga using equation (3). By assuming the atmosphere-ocean system is in steady state, calculate the necessary 4.1 Ga seafloor weathering flux to ensure outgassing + weathering inputs equal burial outputs. Discard parameter combinations that require negative seafloor weathering fluxes at 4.1 Ga.

Step 7: Calculate coefficients in expressions relating carbonate saturation to carbonate precipitation fluxes. Note that the solid Earth need not necessarily be in steady state initially i.e. balance between mantle and crustal fluxes is not enforced.

Step 8: Now that all initial carbon and alkalinity fluxes are known, initial isotope fluxes are calculated assuming at 4.1 Ga all reservoirs are isotopically homogeneous (negligible organic carbon).

Text S4: Subduction efficiency

This section describes the parameterization used to incorporate subduction efficiencies for carbonate and organic carbon. As noted in the main text, the subduction efficiency of organic carbon, ξ_{org}, is assumed to be constant over Earth history with a broad prior range from 0.2-0.8 (Duncan & Dasgupta 2017). To calculate the time-evolution of carbonate subduction efficiency, ξ_{carb}, we assume the fraction of carbonates that reach the mantle is approximated by the following expression (Höning et al. 2019):

$$\xi_{carb} = \frac{c_{crit} - 0.3}{0.6 - 0.3} \qquad (12)$$

Here, the critical temperature gradient ratio, c_{crit}, is related to mantle temperature and surface temperature as follows (Höning et al. 2019):

$$c_{crit} = \frac{\left(3.125\times10^{-3}\times80 + 835.5\right) - T_S}{T_{mantle} - T_S} \qquad (13)$$

The term in the brackets is the decarbonation temperature at 80 km depth. The typical mantle temperature at subduction zones, T_{mantle} (K), can be approximately related to relative internal heatflow using the following parameterization:

$$T_{mantle} = T_{mantle}^{modern} \times Q^{0.1} \tag{14}$$

This ensures a plausible range of mantle temperatures at 4.1 Ga, around 1500-2200 K. The modern mantle subduction zone temperature, T_{mantle}^{modern} (K), is an unknown parameter representing average subduction zone conditions, with a prior ranging from 1300 – 1800 K. This range was chosen because it corresponds to an appropriate range (0.2-0.8) in the modern carbonate weathering subduction efficiency, $\xi_{carb}(modern)$. Modern runs where $\xi_{carb}(modern) > \xi_{org}$ were excluded.

Text S5: Carbon mass balance and the rock cycle

The system of equations that govern the time-evolution of reservoirs (eq. (2)) and isotopic abundances (eqs. (20)-(23)) guarantee that total carbon and ^{13}C are conserved. Losses from surface reservoirs are necessarily balanced by crustal and mantle gains, and vice versa. Mass conservation is illustrated in Fig. S7, which shows outputs from a typical nominal model instance. Fig. S7a demonstrates that total carbon is conserved over Earth history, i.e. $R_{AO} + R_{org} + R_{carb} + R_{mantle}$ = constant. Fig. S7b shows that ^{13}C is conserved over Earth history, i.e. $\delta^{13}\mathrm{C}_{\mathrm{AO}} R_{AO} + \delta^{13}\mathrm{C}_{\mathrm{carb}} R_{carb} + \delta^{13}\mathrm{C}_{\mathrm{org}} R_{org} + \delta^{13}\mathrm{C}_{\mathrm{mantle}} R_{mantle}$ = constant. The remaining subplots show how outgassing, weathering, and burial fluxes are co-evolving with these reservoirs.

While our model conserves carbon mass and isotopes, it is a simplification of the real rock cycle because we do not separately track continental and marine reservoirs. Instead, continental and marine carbon are combined into a single crustal reservoir. Fluxes from (to) the continents or seafloor into (from) the atmosphere-ocean are separately parameterized such that they can respond to changing surface conditions (e.g. climate, subaerial land fraction, ocean pH) in a plausible way, but all weathering and burial fluxes are adding or subtracting carbon from the same well-mixed crustal reservoir, exchanging carbon and alkalinity with the atmosphere-ocean system.

To what extent does this simplification of the rock cycle impact our results? In the real rock cycle, detrital organic carbon may be transferred from continental reservoirs to marine sediments. Because all crustal organic carbon is represented by a single “box” in our simplified model, detrital organic carbon

fluxes need not be explicitly modeled as they do not affect the surface redox budget or carbon cycle. The organic burial and weathering fluxes in our model represent autochthonous organic burial, and chemical oxidation of organics, respectively. The observed carbon isotope record is some combination of autochthonous and allochthonous organic carbon. In our model, we assume that the autochthonous component dominates, which is a potential oversimplification.

However, if we make the opposite endmember assumption, that the carbon isotope record at any given time is not determined by instantaneous burial fluxes but rather by the average isotopic composition of the entire crustal organic reservoir (i.e. maximum detrital recycling), then the change in model outputs is minimal. Fig. S8 compares the evolution of the carbon isotope record as calculated by our model using these two detrital recycling assumptions (and both are compared to the real carbon isotope record). Fig. S8a is identical to Fig. 2a in the main text, which effectively assumes all organic burial is autochthonous with no recycled component. Fig. S8b recalculates the carbon isotope record in our model assuming the observed record at any given time is the average of the entire reservoir previously deposited, as opposed to the ^{13}C of buried organic carbon at that moment. The differences in outputs between the two approaches are minor. Maximizing detrital recycling results in a more gradual transitions at the Archean-Proterozoic and Proterozoic-Phanerozoic boundaries as it takes a few hundred million years for the reservoirs to adjust to changing boundary conditions. Corresponding differences between the apparent fractional organic burial histories (Fig. S8c and S8d) are similarly minor.

Separately tracking the evolution of continental and seafloor reservoirs of carbon would be challenging given that both the tectonic regime and the growth of continents in the Archean are highly uncertain; partitioning carbon between these two reservoirs would be largely guesswork. However, by permitting weathering and burial fluxes to be modulated by the uncertain growth in continents, we already allow for a broad range of crustal carbon growth histories. Fig. S9 shows 100 random outputs from our nominal model (Fig. 2 and Fig. 3 in the main text), where the growth in the continental carbonate reservoir is plotted on a linear scale. The sometimes-rapid crustal carbon accumulation in Fig. S9 reflects the few hundred million years it takes for the model to spin up to a quasi-steady state, where reservoir-dependent sinks of carbon balance outgassing fluxes.

Supplementary Figures

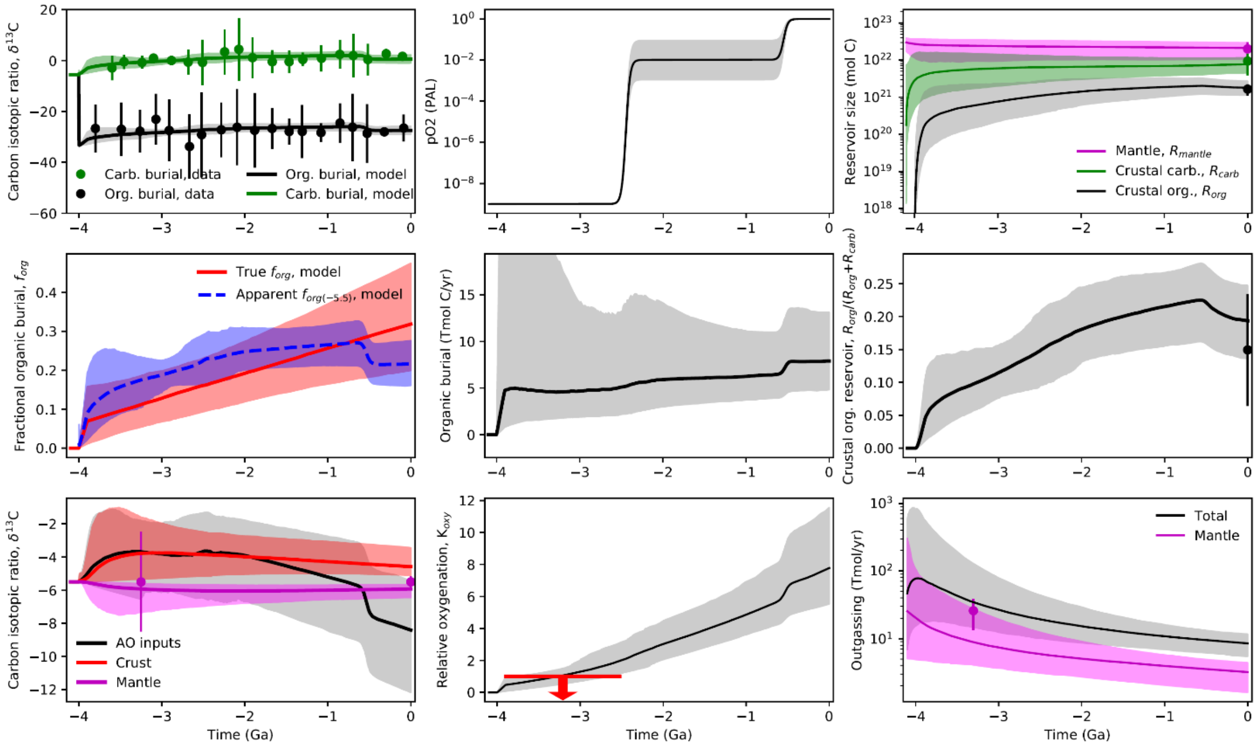

Fig. S1: Linear fractional organic burial parameterization. This figure is identical to Fig. 2 in the main text except the inverse analysis was repeated using a linear parameterization for fractional organic burial over Earth history (with unknown gradient and intercept). The inferred change in fractional organic burial over Earth history is essentially the same as when the three-component organic burial parameterization is used, and the fit to geologic constraints is equivalent.

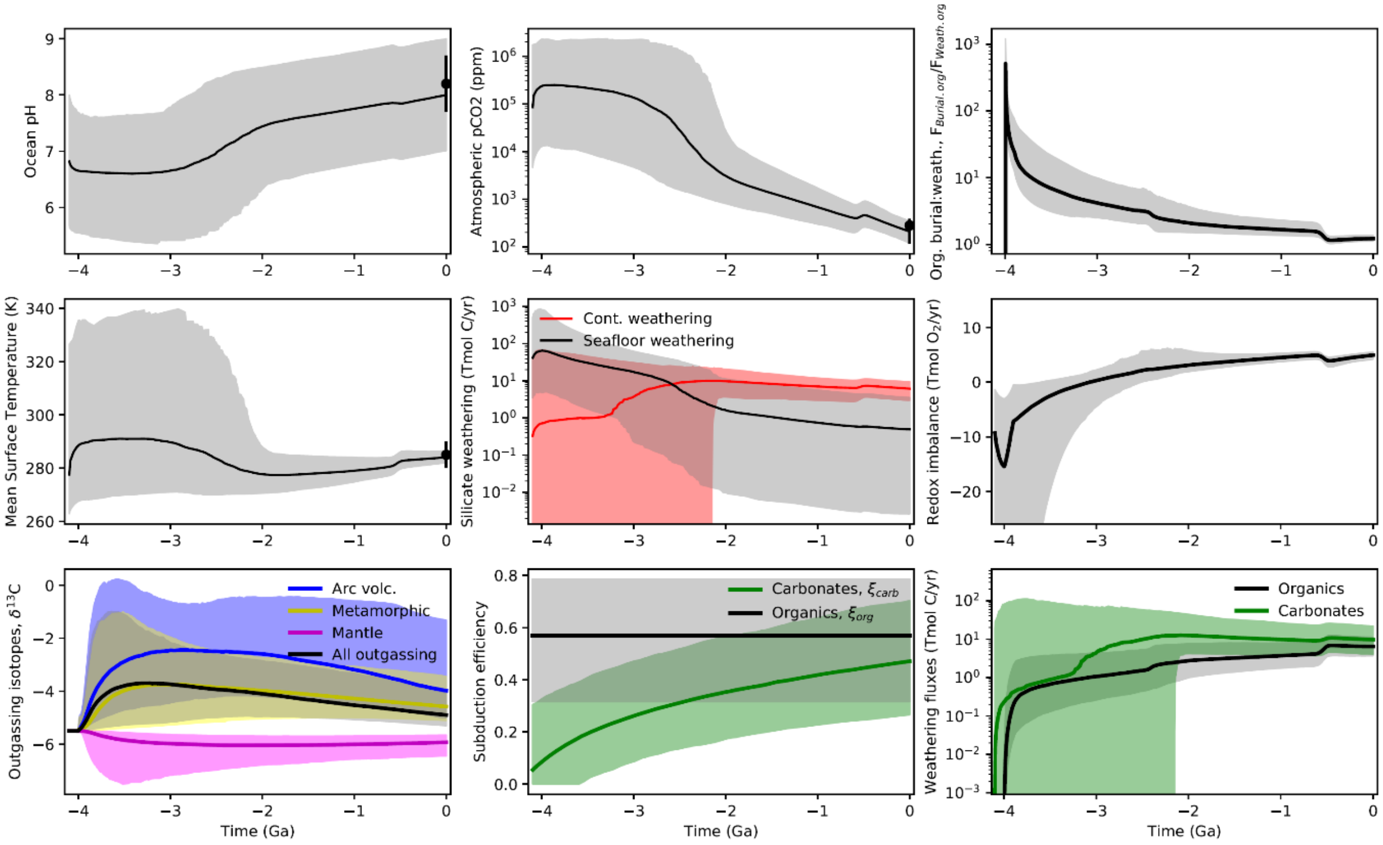

Fig. S2: Additional carbon cycle variables for linear fractional organic burial parameterization. This figure is identical to Fig. 3 in the main text except the inverse analysis was repeated using a linear parameterization for fractional organic burial over Earth history (with unknown gradient and intercept).

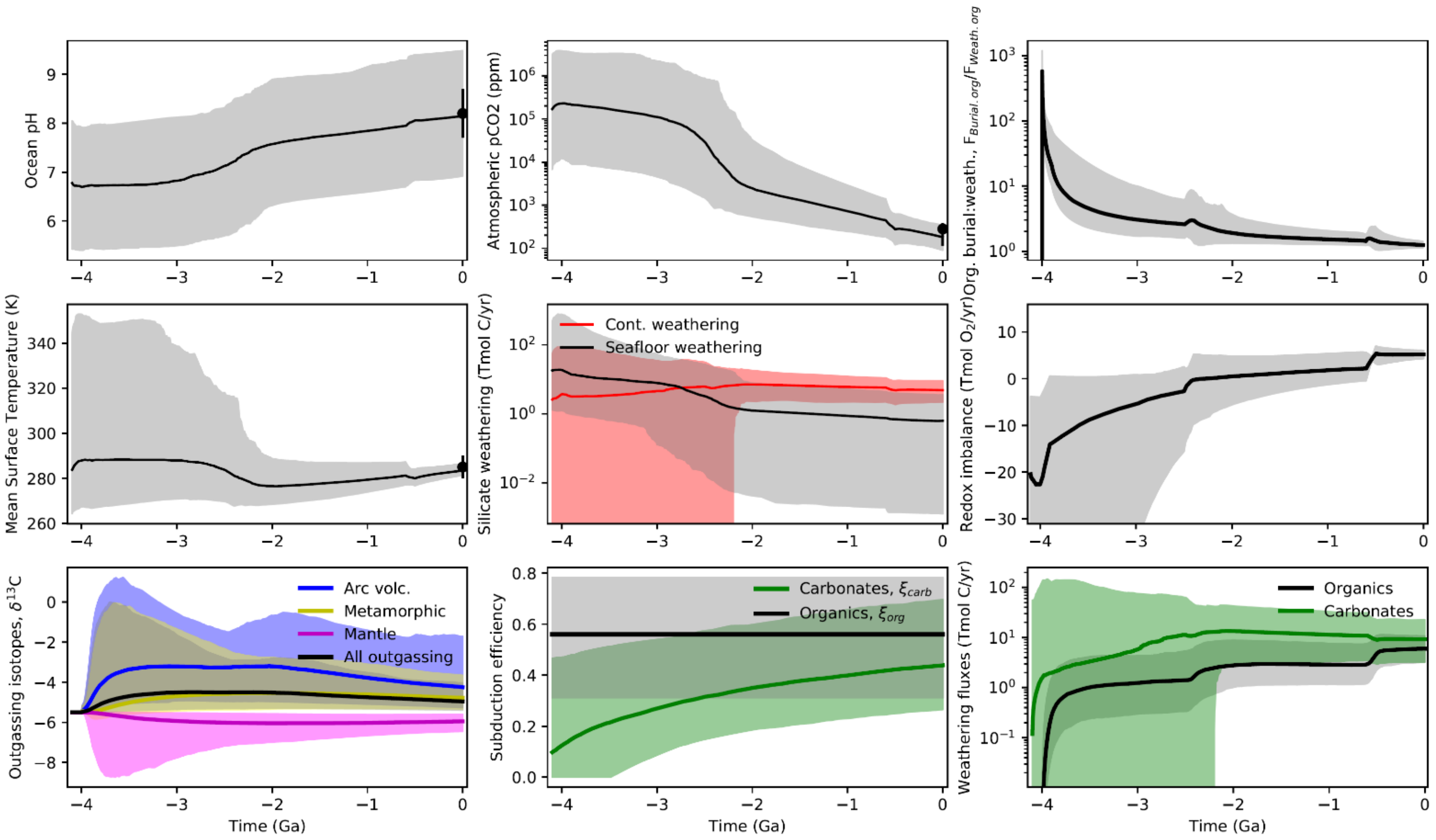

Fig. S3: Additional carbon cycle variables for reduced mantle scenario. This figure is identical to Fig. 3 in the main text except the inverse analysis was repeated assuming a reduced Archean mantle.

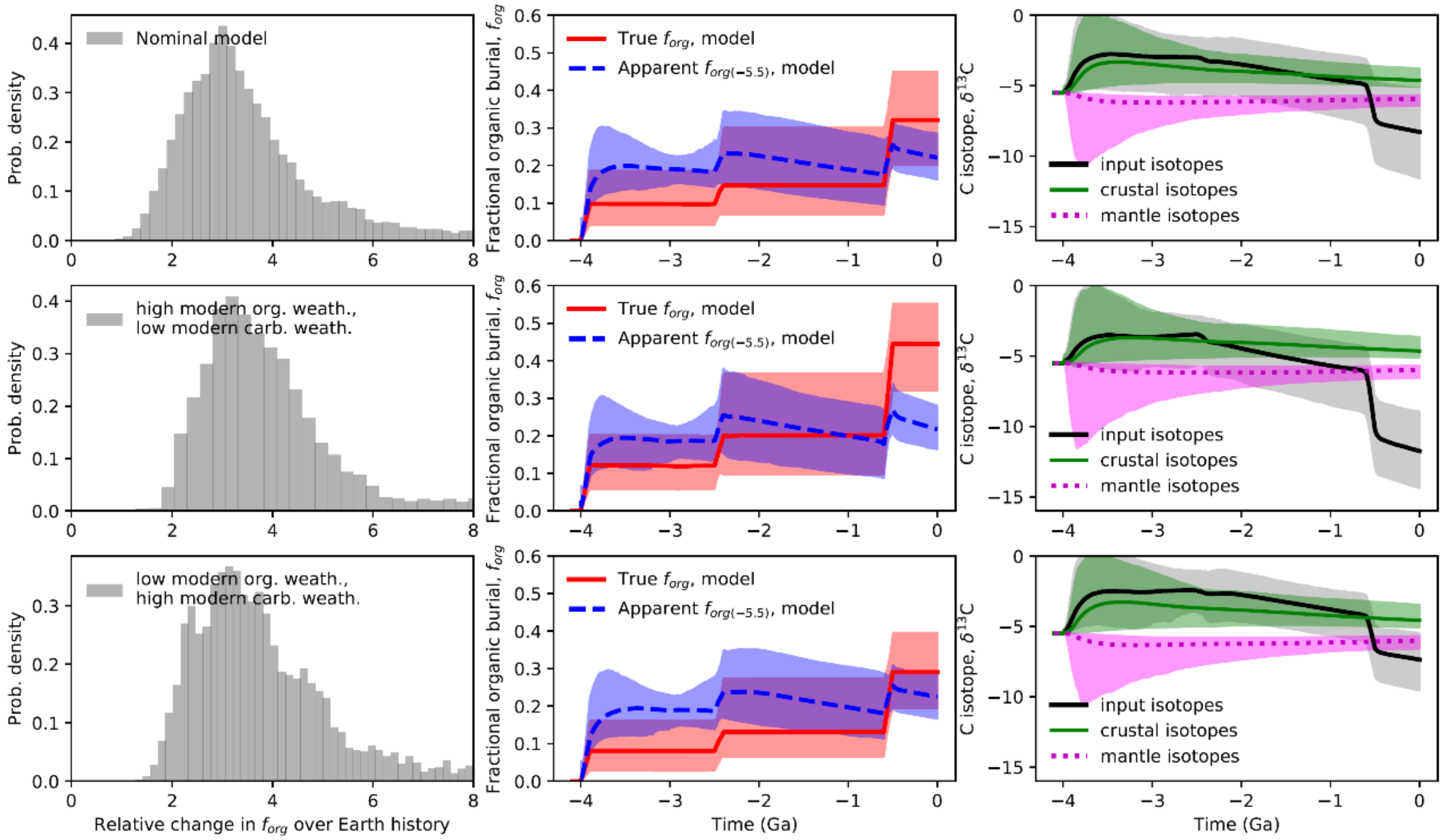

Fig. S4: Sensitivity of results to modern flux assumptions. First row shows results from nominal model, ($F_{\text{oxid}}^{modern} + F_{\text{thermo}}^{modern}$ = 3-9 Tmol C/yr, $F_{\text{Weath\_carb}}^{modern}$ = 7-25 Tmol C/yr), second row shows results assuming a high range for the modern organic weathering flux and a low range for the modern carbonate ($F_{\text{oxid}}^{modern} + F_{\text{thermo}}^{modern}$ = 6-12 Tmol C/yr, $F_{\text{Weath\_carb}}^{modern}$ = 7-15 Tmol C/yr), and third row shows results assuming a low modern organic weathering flux and a high modern carbonate weathering flux ($F_{\text{oxid}}^{modern} + F_{\text{thermo}}^{modern}$ = 3-9 Tmol C/yr, $F_{\text{Weath\_carb}}^{modern}$ = 15-25 Tmol C/yr). The relative change in f_{org} over Earth history (first column) is insensitive to assumed modern fluxes. However, the absolute value of modern fractional organic burial does depend strongly on modern fluxes; high modern organic weathering in combination with low carbonate weathering would imply modern f_{org} around 0.4. This would also imply isotopically light carbon inputs into the atmosphere-ocean system (third column), which may conflict with empirical constraints (see main text).

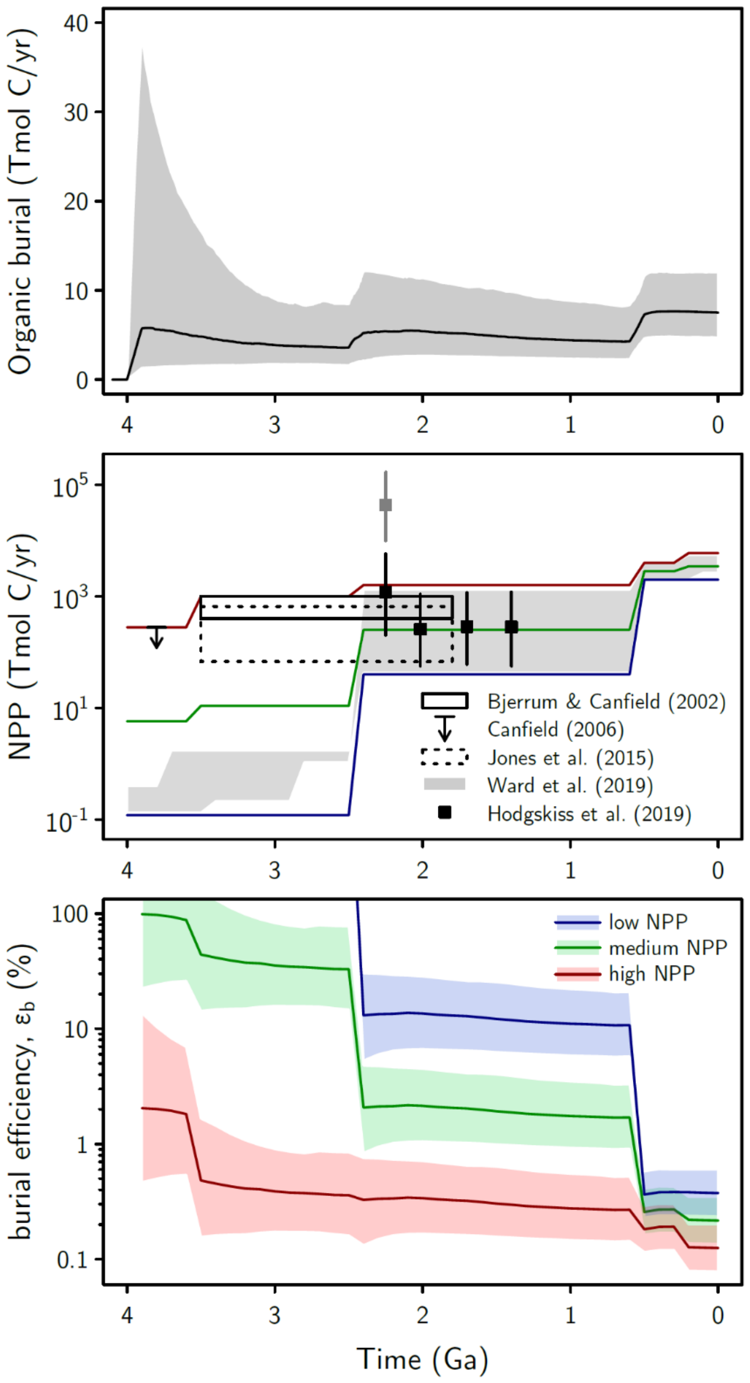

Fig. S5: Burial efficiency through time for reduced Archean mantle scenario. This figure is identical to Fig. 8 in the main text except the calculations are done for the reducing Archean mantle scenario (Fig. 6).

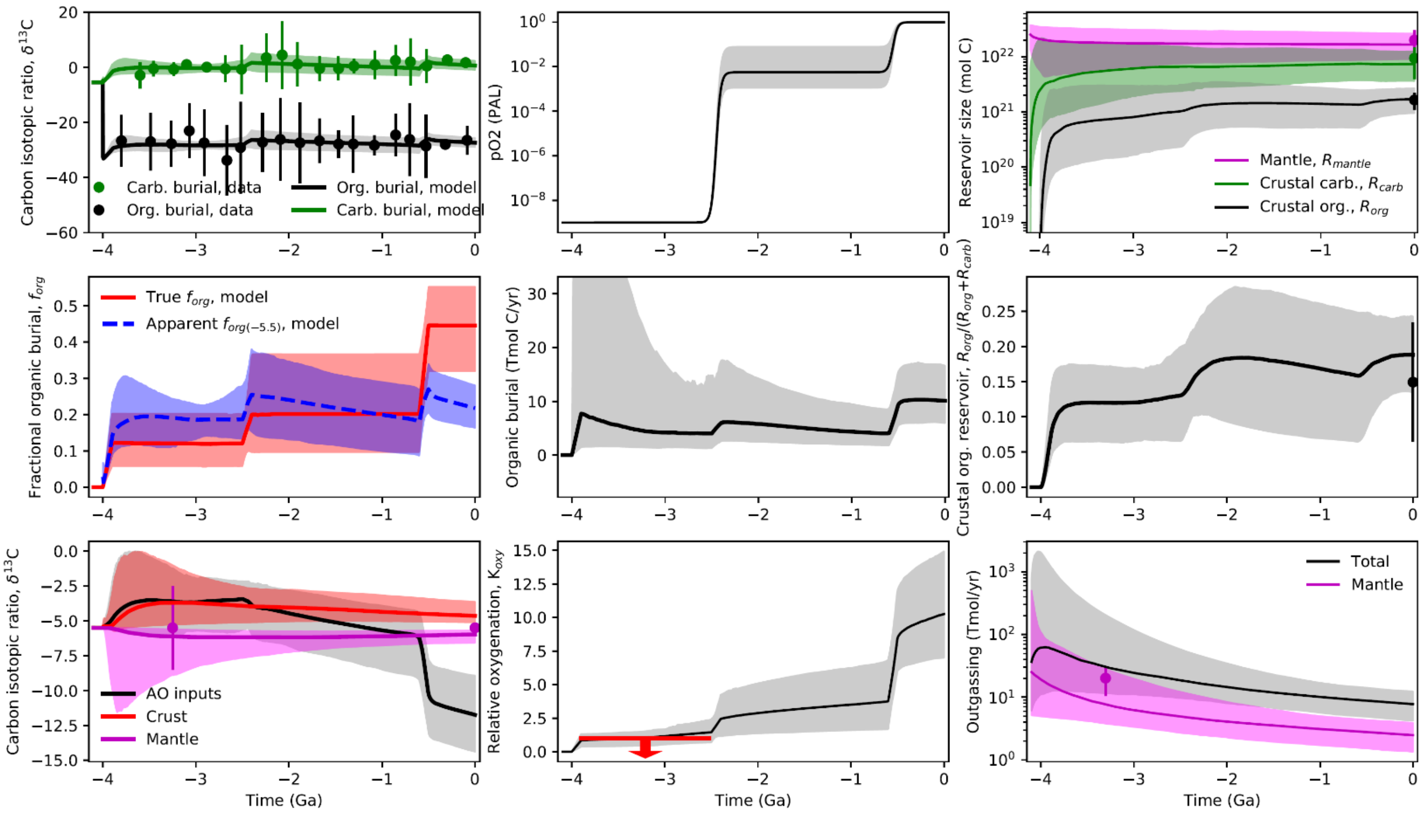

Fig. S6: Carbon cycle outputs for sensitivity test with high modern organic weathering and low modern carbonate weathering (high modern fractional organic burial).

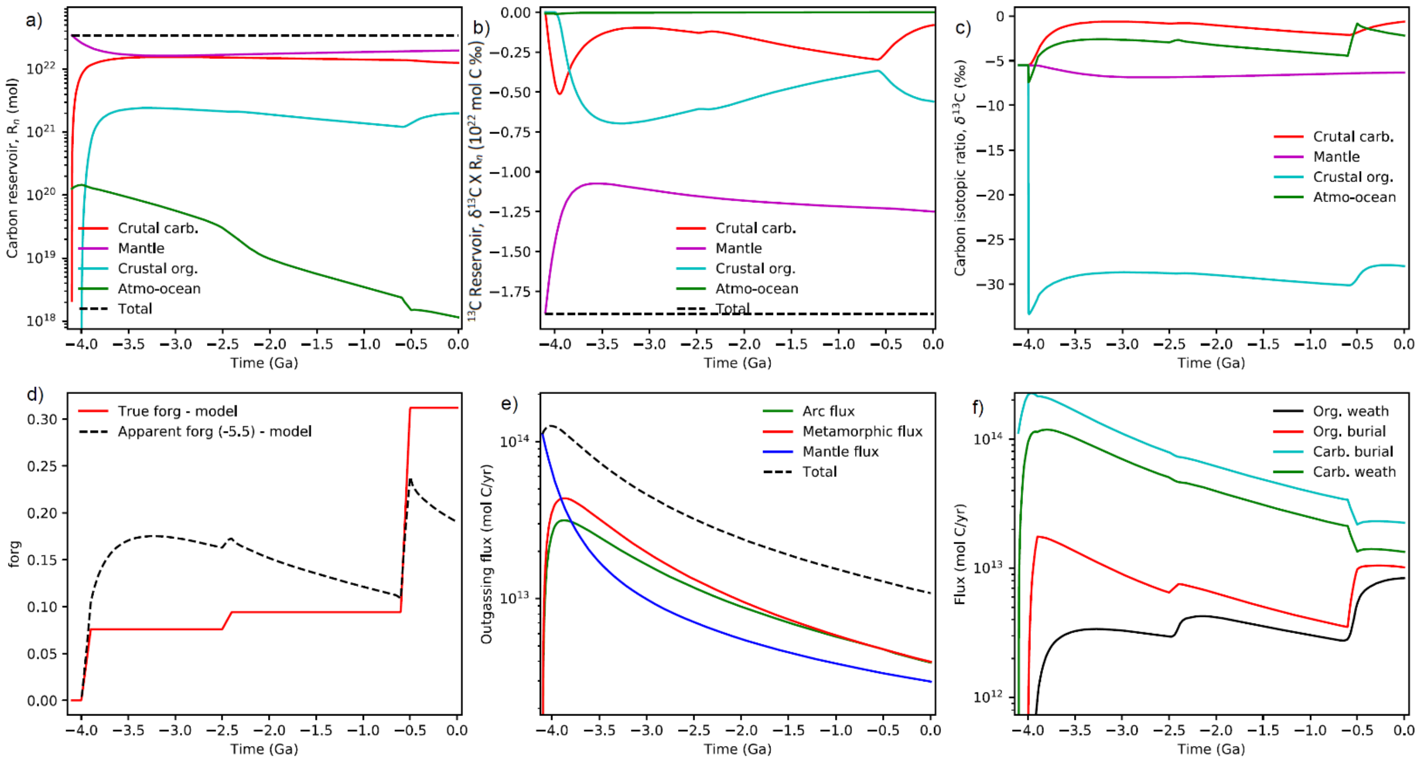

Fig. S7: Demonstration of mass conservation. Outputs are shown for a typical model run using our nominal model (Fig. 2 and 3 in the main text). Subplot (a) shows the conservation of total carbon (black dashed line), whereas subplot (b) shows the conservation of ^{13}C (black dashed line). Specifically, we show that $\delta^{13}\mathrm{C} \times R_n$ is conserved, where R_n is the relevant reservoir in mol C and *n* = crustal carbonates, crustal organics, mantle, or atmosphere-ocean. The time-evolution of reservoir isotope abundances (c), fractional organic burial (d), outgassing fluxes (e), and weathering and burial fluxes (f) are also plotted for comparison.

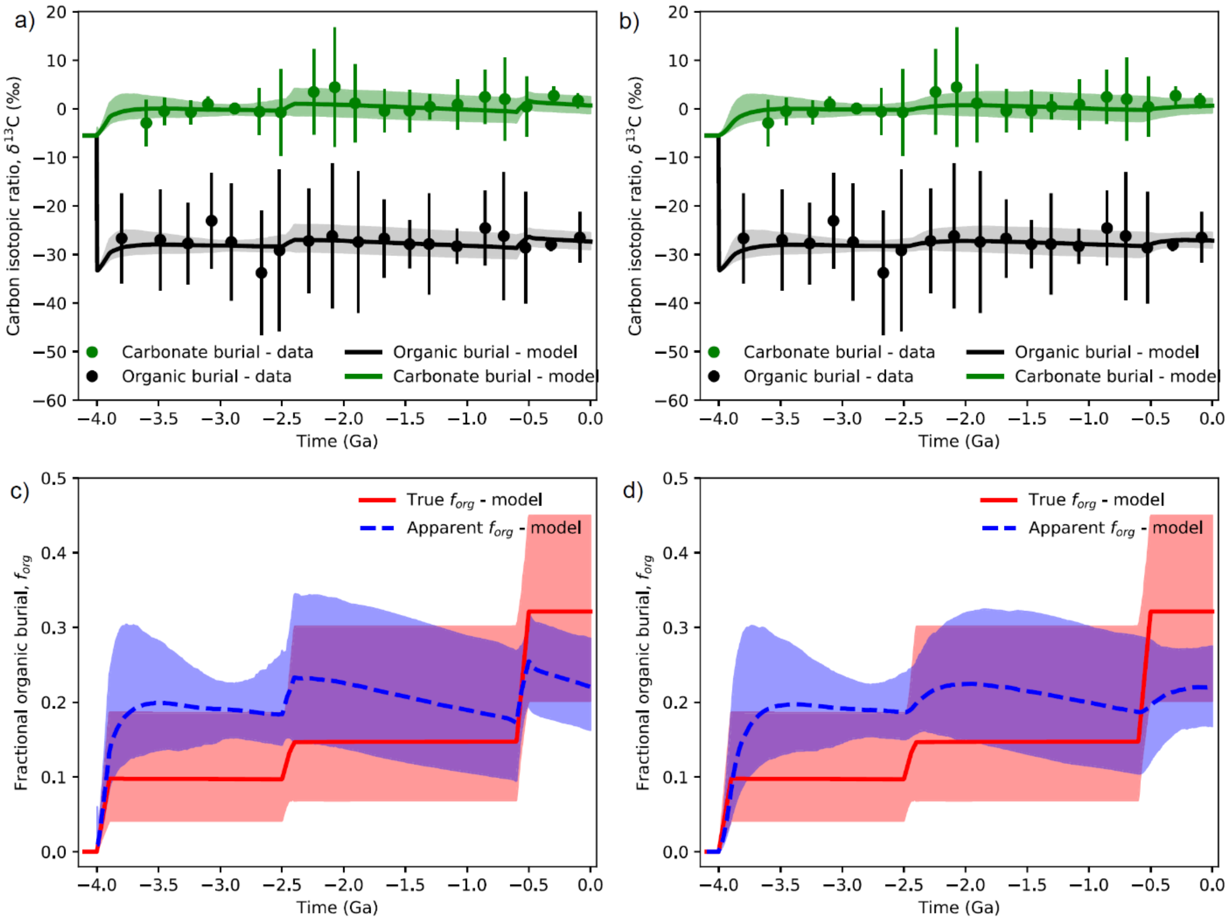

Fig. S8: Impact of detrital recycling assumptions. Subplot (a) shows a comparison between our model and data with the nominal assumption that the observed isotope record is 100% autochthonous organic carbon and no recycled detrital carbon (identical to Fig. 2a in the main text). Subplot (b) recalculates the model isotope record while making the opposite assumption that the carbon isotope record at any given time is composed of the well-mixed average of all previously deposited organic carbon. Subplot (c) shows the apparent fractional organic burial through time using the model outputs in (a), whereas subplot (d) shows the apparent fractional organic burial through time using the model outputs in (b). The difference in the evolution of the carbon isotope and apparent organic burial records between the two approaches are minor. Maximizing detrital recycling results in a more gradual transitions at the Archean-Proterozoic and Proterozoic-Phanerozoic boundaries as it takes a few hundred million years for the reservoirs to adjust to changing boundary conditions.

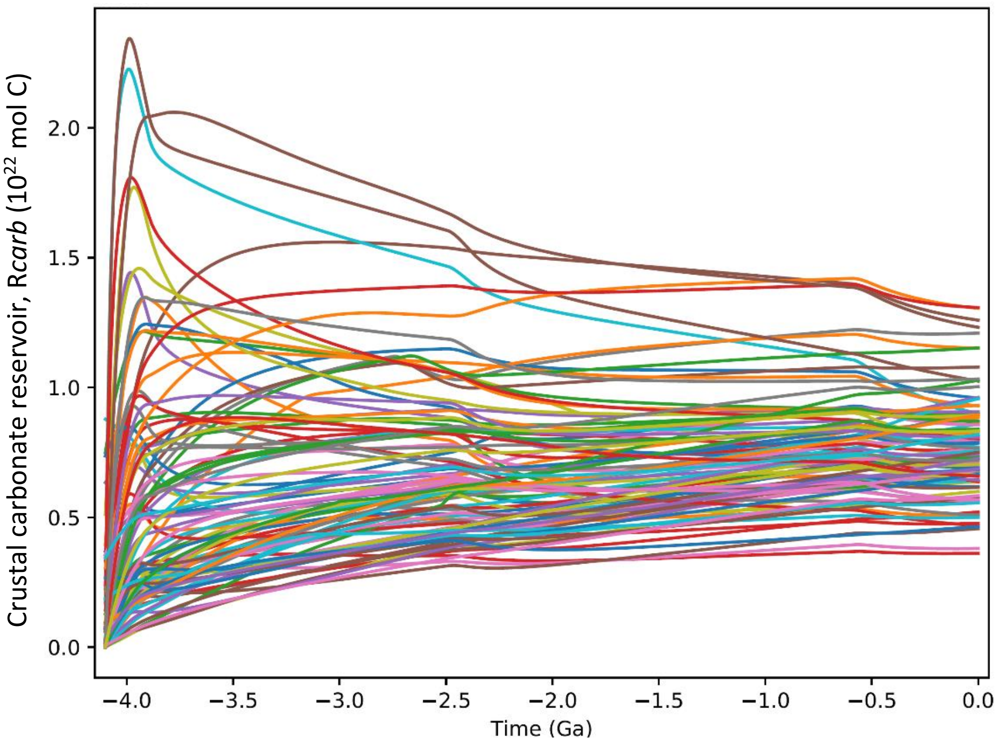

Fig. S9: The growth of the continental carbonate reservoir. This figure shows 100 outputs from our nominal model illustrating the growth of the continental carbonate reservoir, R_{carb} (c.f. Fig. 2c). Broad parameterizations for continental and seafloor weathering fluxes, and a wide assumed range of land fraction evolutions, permit a range of rapid and gradual growth curves.

Supplementary References

Duncan, M. S., & Dasgupta, R. (2017). Rise of Earth's atmospheric oxygen controlled by efficient subduction of organic carbon. *Nature Geoscience, 10*(5), 387.

Höning, D., et al. (2019). Carbon cycling and interior evolution of water-covered plate tectonics and stagnant lid planets. *arXiv preprint arXiv:1905.12392*.

Krissansen-Totton, J., et al. (2018). Constraining the climate and ocean pH of the early Earth with a geological carbon cycle model. *Proceedings of the National Academy of Sciences*, 201721296.

Krissansen-Totton, J., et al. (2015). A statistical analysis of the carbon isotope record from the Archean to Phanerozoic and implications for the rise of oxygen. *American Journal of Science, 315*(4), 275-316.

Krissansen-Totton, J., & Catling, D. C. (2017). Constraining climate sensitivity and continental versus seafloor weathering using an inverse geological carbon cycle model. *Nature communications, 8*, 15423.
